# 1,2,3-Triazole framework: a strategic structure for C–H⋯X hydrogen bonding and practical design of an effective Pd-catalyst for carbonylation and carbon–carbon bond formation†

**DOI:** 10.1039/d1ra03356e

**Published:** 2021-06-11

**Authors:** Fatemeh Mohammadsaleh, Maryam Dehdashti Jahromi, Abdol Reza Hajipour, Seyed Mostafa Hosseini, Khodabakhsh Niknam

**Affiliations:** Department of Chemistry, Faculty of Nano and Bio Science and Technology, Persian Gulf University Bushehr Iran f.mohammadsaleh@gmail.com niknam@pgu.ac.ir; Faculty of Engineering, Jahrom University Jahrom Iran; Pharmaceutical Research Laboratory, Department of Chemistry, Isfahan University of Technology Isfahan 84156 Islamic Republic of Iran; Department of Pharmacology, University of Wisconsin, Medical School, 1300 University Avenue Madison 53706-1532 WI USA

## Abstract

1,2,3-Triazole is an interesting N-heterocyclic framework which can act as both a hydrogen bond donor and metal chelator. In the present study, C–H hydrogen bonding of the 1,2,3-triazole ring was surveyed theoretically and the results showed a good agreement with the experimental observations. The click-modified magnetic nanocatalyst Pd@click-Fe_3_O_4_/chitosan was successfully prepared, in which the triazole moiety plays a dual role as both a strong linker and an excellent ligand and immobilizes the palladium species in the catalyst matrix. This nanostructure was well characterized and found to be an efficient catalyst for the CO gas-free formylation of aryl halides using formic acid (HCOOH) as the most convenient, inexpensive and environmentally friendly CO source. Here, the aryl halides are selectively converted to the corresponding aromatic aldehydes under mild reaction conditions and low Pd loading. The activity of this catalyst was also excellent in the Suzuki cross-coupling reaction of various aryl halides with phenylboronic acids in EtOH/H_2_O (1 : 1) at room temperature. In addition, this catalyst was stable in the reaction media and could be magnetically separated and recovered several times.

## Abstract 2

The C–H hydrogen bonding of a 1,2,3-triazole framework was studied. An Fe_3_O_4_–chitosan core–shell incorporating a triazole/Pd complex was investigated as a nanocatalyst in carbonylation and C–C coupling.

## Graphical abstract

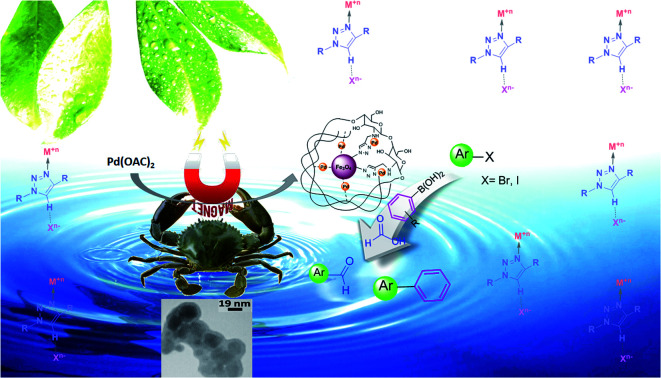

## 1. Introduction

Nowadays, new advances in the chemical synthesis and catalysis areas are strongly tied to green chemistry. The goal is the utilization of environmentally friendly reagents and a set of principles that minimize or eliminate contamination and pollution in the design of new chemical processes. Many efforts have been directed towards the development of bio-based materials in the synthesis routes due to the limited availability of fossil fuel and growing environmental concerns.^1,2^

Academic and industrial discoveries of efficient and selective catalysts for a wide variety of organic reactions have had a huge influence on minimizing the costs of manufacturing and waste disposal.^3^ Heterogenization of homogeneous catalysts on solid supports has received significant attention in the catalysis researches and follows the majority of the principles of the green chemistry framework in synthesis routes in terms of the separation and catalyst-recycling.^4,5^ Therefore, the design and development of a suitable support as catalyst carrier capable of improving the catalyst characteristics, especially for metal-based catalysts, by a cooperative effect between the metal complex and the support, is still an important challenge for successful catalysts.^6^

**Scheme 1:**
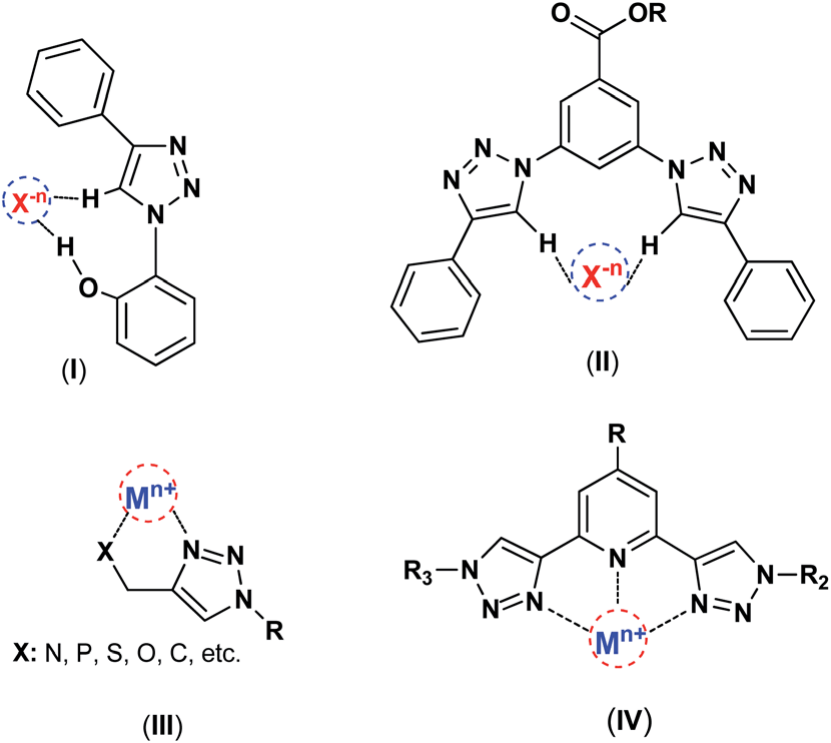
The interactions of 1,2,3-triazole framework with metal ions and anions, (I),^16^ (II),^9^ (III),^13,17^ (1V).^13^

Chemistry is knowledge of wonders, and in the depths of this science, marvelous mysteries can be discovered. 1,2,3-Triazoles are interesting class of N-heterocyclic compounds, which are typically prepared by Cu(i)-catalyzed azide–alkyne 1,3-dipolar cycloaddition (CuAAC) reaction recognized as a highly important example of “click chemistry”.^7,8^ 1,2,3-Triazole produced in the click reaction offers properties beyond just the sum of its components, the azide and alkyne. It has shown various successful applications in different domains such as anion recognition,^9–11^ catalysis.^12,13^ surface modification and material science.^14,15^ This nitrogen-rich aromatic entity has a great dipole moment (∼5 D), and can act as the metal chelator and C–H hydrogen bond donor interacting with electron-rich partners such as anions (Scheme 1).

Several research groups have reported 1,4-disubstituted-1,2,3-triazoles as appropriate host for the complexation of different anions and have employed them in anion recognition where many weak C–H⋯X^*n*−^ interactions were accrued through hydrogen bonding and electrostatic interactions.^10,11^ Indeed, the large dipole moment of 1,2,3-triazoles and extrinsic polarization of a carbon atom in the 5-position afford hydrogen bonds involving C–H groups. Apart from anion complexation as hydrogen-bond-donor, the nitrogen-rich 1,2,3-triazols have the ability to coordinate a metal center as the N-donor ligands.^8,18^ Such characteristics introduce triazoles as important and powerful candidates in the catalysis field, particularly in metal-based catalysts. The 1,2,3-triazole as a high stable linker have attracted much attention for surface modifications and catalyst immobilization, and as the effective N-donor ligand can coordinate with metal ions and stabilize catalytic species on the surface of solid supports.^19–21^ Recently, these units have great tendency to coordination with many metal ions like Cu, Re, Pd, and *etc.*^22,23^

Polysaccharides have been widely applied as a suitable architecture for solid catalysts. Chitosan (CS), the *N*-deacetylated derivative of chitin, is a sample of such polysaccharides that is widely spread in living organisms. Due to the presence of reactive functional groups, free-amino groups and hydroxyl groups, CS and its composite-derivatives are considered to be proper solid supports for the immobilization of a metal catalyst. A main limiting problem in the consuming of CS in the catalysis fields is poor chemical resistance and mechanical strength, which significantly reduces the recycle life of the catalysts based on this biopolymer. Accordingly, it is often necessary to use stronger ligands or metal/metal oxide nanoparticles to modify fresh CS properties. Physical and chemical modifications improve pore size, mechanical strength, chemical stability, hydrophilicity and biocompatibility of the CS-based composites for catalytic applications. Several studies have been conducted toward developing chitosan coated magnetic nanoparticles. Miserez *et al.*^24^ prepared a composite of catechol-functionalized chitosan with superparamagnetic iron oxide (γ-Fe_2_O_3_) nanoparticles that represented a significant improvement in functionality of chitosan-based biomaterials. Zhao *et al.*^25^ reported a mesostructured Fe_3_O_4_/chitosan composite as a pH-responsive drug-delivery system. Movassagh *et al.*^26^ have reported magnetic porous chitosan-thienyl imine palladium(ii) complex as an efficient and magnetically recoverable catalyst for the Mizoroki–Heck reaction.

Magnetic nanoparticles have been a topic of great interest in recent years, as their characteristics make them appropriate for use in various fields such as physics, medicine, biology, materials and catalysis science.^27,28^ The advantage of magnetic catalysts is the convenient separation and recovery from the reaction mixture using an external magnet. However, Fe_3_O_4_ nanoparticles have a tendency to aggregate because of strong magnetic dipole–dipole attractions between particles. Coating Fe_3_O_4_ nanoparticles with inorganic/organic polymers increases the stability of Fe_3_O_4_-based catalysts in reaction medium.

**Scheme 2:**
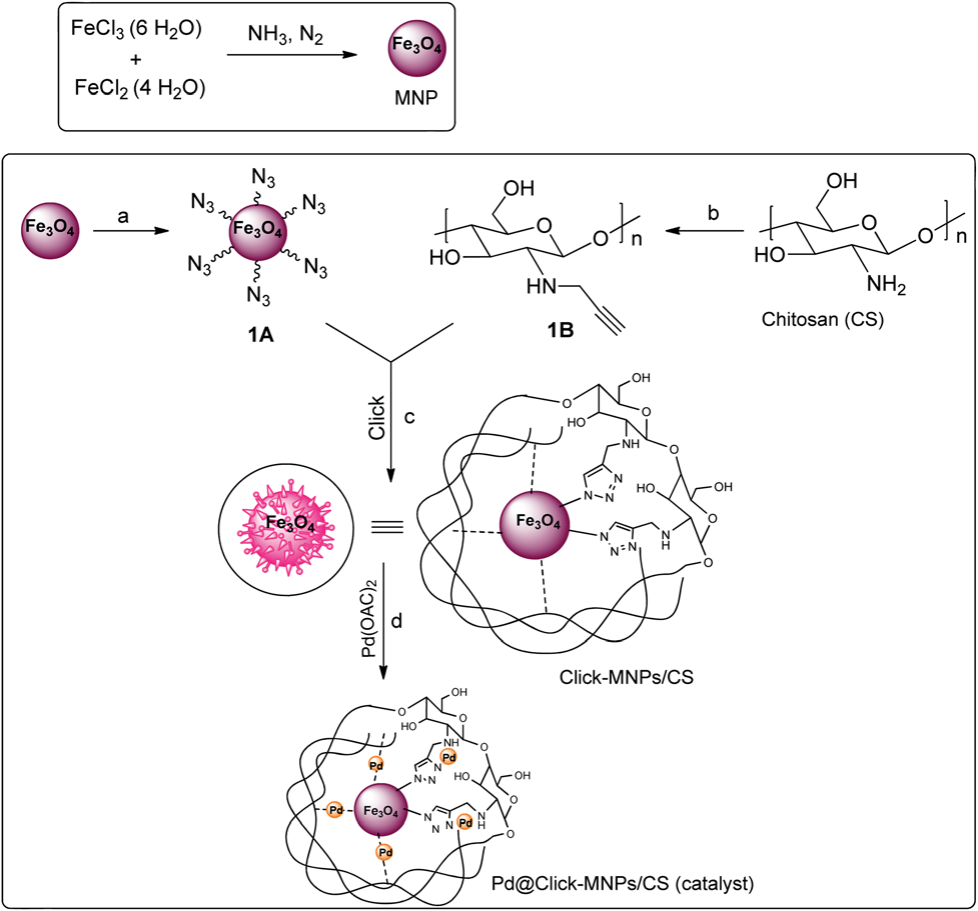
Synthesis of Pd@click-Fe_3_O_4_/chitosan catalyst. Reaction conditions: (a) 3-azidopropyltrimethoxysilane, ethanol, 40 °C, 18 h, N_2_; (b) propargyl bromide, DMF, 60 °C, 24 h; (c) Cu_2_O, H_2_O/DMF, 60 °C, 3 d; (d) toluene, Pd(OAc)_2_, r.t., 2 d.

In continuation of our efforts towards the design of new and versatile catalysts employing the 1,2,3-triazole frameworks,^17,29–31^ in this work, the metal coordination ability of 1,2,3-triazole ring was used for surface modification and new catalyst design and the triazole-modified core–shell Fe_3_O_4_/chitosan was synthesized as a sustainable solid support for the immobilization of palladium catalyst. Herein, chitosan (CS) was used as an effective stabilizer agent for Fe_3_O_4_ nanoparticles (MNPs), in which polymer chains and Fe_3_O_4_ particles are connected to each other covalently *via* click reaction. The detailed route is shown in Scheme 2. This covalently-clicked connection increases the strength of the resulted solid support, and also the presence of nitrogen-rich triazole unites with excellent chelating ability to metal center could cause an increased capability of metal-immobilization for the support. The incorporation of palladium(ii) complex into the click-Fe_3_O_4_/chitosan (click-MNPs/CS) gave the Pd@click-MNPs/CS, which could be successfully applied as a magnetic heterogonous, efficient, and green catalyst for the formylation of aromatic halides using formic acid as the CO-source in the presence of DCC activator.

The general term ‘carbonylation’ was expressed particularly for the various transformations that incorporate carbon monoxide into the organic compounds. Recently, carbonylation has become a powerful strategy for the preparation of carbonyl-containing organic combinations.^32–34^ To date, many researchers have focused their studies on the new CO gas-free carbonylation systems^35–37^ and reported the various carbonyl sources such as formic acid,^38,39^ Mo(CO)_6_,^40^ oxalic acid^36^ and *N*-formylsaccharin.^41^ Formic acid is an eco-friendly, cheap and available carbonyl source and can generate a carbon monoxide (CO) molecule *in situ* during the reaction process.^42^ The formic acid-assisted carbonylation catalyzed by palladium catalysts^38,39^ is an efficient approach for constructing carbonyl compounds. In another part of this work, the Pd@click-MNPs/CS was employed as a recyclable catalyst in the Suzuki–Miyaura cross-coupling of diverse aryl halides with phenylboronic acids in green solvent at room temperature. We also have studied the hydrogen bonding of triazole C–H by ^1^HNMR analysis of 1-benzyl-4-phenyl-1*H*-1,2,3-triazole in DMSO and CDCl_3_ solvents. In this part, the effects of the solvent and its polarity on the most stable structure of this compound and ^1^HNMR signal of triazole proton in the gas phase, DMSO and in CDCl_3_ solvent were theoretically investigated.

## 2. Results and discussion

### 2.1. Synthesis and characterization of composite

Due to ambivalent character of triazoles, they can act as both an N-donor ligand and hydrogen donor, showing the complexations with both metal cations and anions. 1,2,3-Triazols as the nitrogen-rich heterocycles have developed as interesting candidates for use in transition-metal catalysis. In this part, the novel magnetic catalyst Pd@click-MNPs/CS containing 1,2,3-triazole unit was synthesized through a ‘‘click’’ reaction, in which 1,2,3-triazole moiety acts as a strong linker and a Pd-chelator. 1,2,3-Triazole having several N-donor positions can be strongly coordinated to palladium(ii) ions and stabilize the catalytic sites in the solid catalyst matrix.

#### 2.1.1. Synthesis and characterization of support with 1,2,3-triazole motives

Herein, we have prepared the covalently modified click-MNPs/CS as a triazole-containing stable support for the immobilization of palladium(ii) species.

##### FTIR spectroscopy

**Fig. 1:**
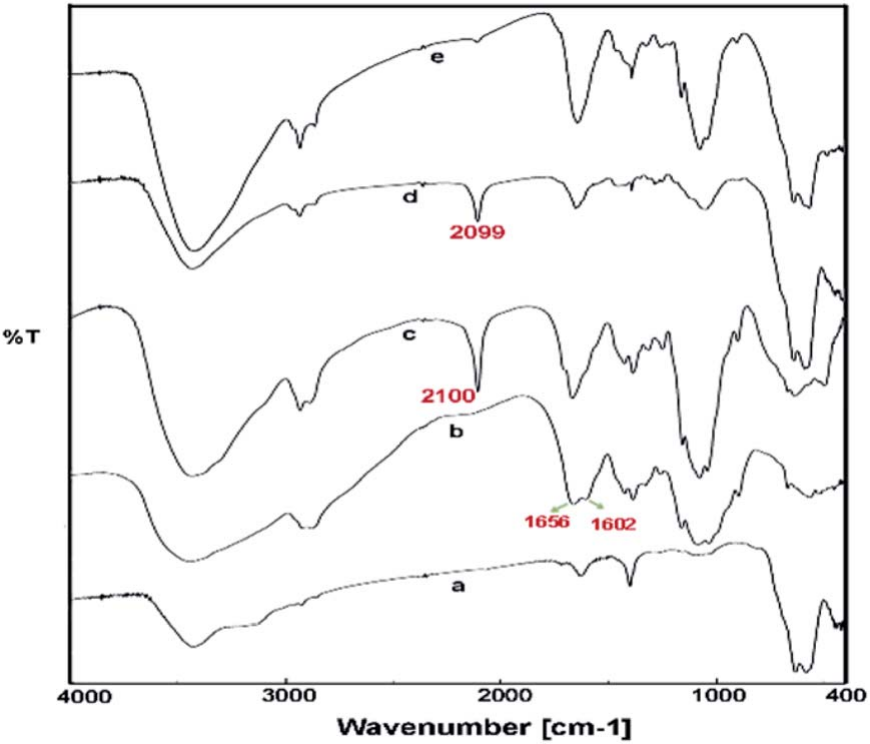
FTIR spectra of Fe_3_O_4_ nanoparticles (MNP) (a), pure chitosan (CS) (b), alkyne-functionalized chitosan (c), azide-functionalized Fe_3_O_4_ (d) and click-MNPs/CS (e).

In the first stage of our strategy, the chitosan and Fe_3_O_4_ were respectively functionalized with alkyne and azide groups. The presence of these groups were confirmed by the appearance of a sharp absorption band at 2100 cm^−1^ in FT-IR spectrum of alkynylated chitosan attributed to the alkyne-groups, and also the N_3_ vibration at 2099 cm^−1^ in spectrum of azide-functionalized Fe_3_O_4_ nanoparticles (Fig. 1c and d). In addition, the intensity of the band at 1602 cm^−1^ (N–H bending vibration) is decreased in the spectrum of the alkynylated chitosan material compared to the pure chitosan spectrum; which confirms that alkyne group has been attached into the amine groups of chitosan after the reaction between chitosan and propargyl bromide.

In the next step, the resulting azide-functionalized Fe_3_O_4_ particles were treated with alkynylated chitosan in the presence of Cu_2_O catalyst in mixed solvent H_2_O/DMF. During the “click” reaction, alkyne sections of alkynylated-CS react with azide groups grafted on the surface of Fe_3_O_4_ particles to obtain the 1,2,3-triazole functionalized-MNPs/CS. The cycloaddition reaction of alkyne and azide groups by the click process was confirmed using FTIR analysis, which revealed disappearance of the absorption bands at 2100 cm^−1^ and 2099 cm^−1^ attributed to the alkyne sections and N_3_, respectively (Fig. 1e). In addition, the IR absorption spectrum of click-MNPs/CS (Fig. 1d) reveals characteristic bands of both species, *i.e.*, Fe_3_O_4_ particles^43,44^ and chitosan (CH, CO, CN, and OH stretching), confirming successful attachment of Fe_3_O_4_ nanoparticles with chitosan backbone.

#### 2.1.2. Synthesis and characterization of Pd/support sample

The resulting click-MNPs/CS was further treated with Pd(OAc)_2_ in toluene at room temperature to afford the desired solid catalyst Pd@click-MNPs/CS, meanwhile, the Pd loading measured by inductively coupled plasma atomic emission spectrometry (ICP-AES) was obtained 1.16%. Also, the Fe content of catalyst was 23.72% as analyzed by the ICP technique.

##### XRD

**Fig. 2:**
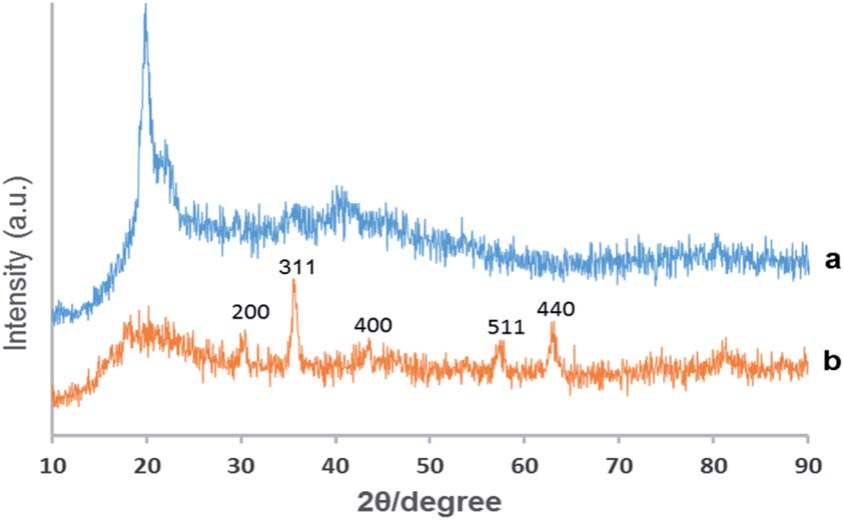
XRD patterns of virginal chitosan (a) and Pd@click-MNPs/CS (b).

Chemical phase analysis of the as-prepared material was performed by X-ray reflective diffraction (XRD) and Fig. 2 exhibits the XRD patterns of virginal CS and the catalyst Pd@click-MNPs/CS. The XRD-diffractions of Fe_3_O_4_ nanoparticles are clearly distinguishable and in good agreement with the crystalline Fe_3_O_4_ particles reported in literatures.^18,45^ Additionally, the broad peak from 2*θ* = 15° to 25° is consistent with an amorphous chitosan polymer. No characteristic peaks for metallic Pd particles are detected which shows the construction of Pd(ii)-complexes in the matrix of the click-MNPs/CS composite.

##### FESEM

**Fig. 3:**
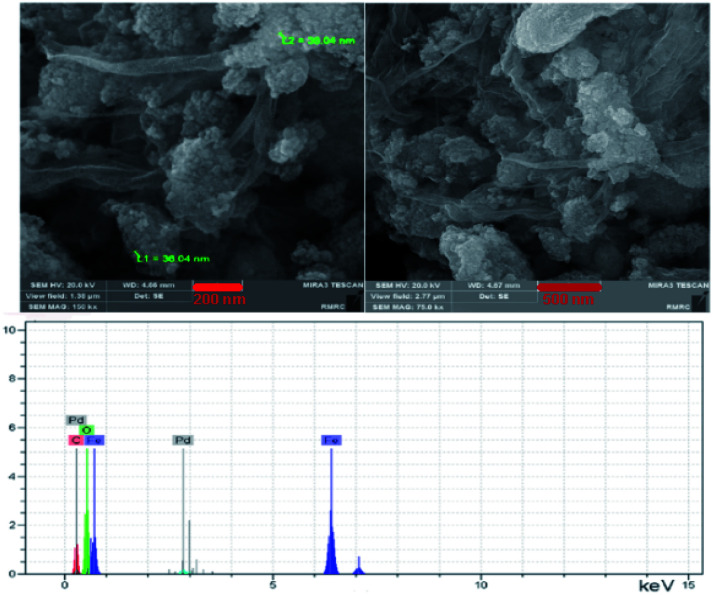
FE-SEM and SEM-EDX of Pd@click-MNPs/CS.

Fig. 3 displays the field emission scanning electron microscopy (FE-SEM) images of the catalyst Pd@click-MNPs/CS. The FE-SEM micrographs clearly show polymer section and Fe_3_O_4_ particles which have been distributed in the polymer matrix. By the SEM-EDX analysis the presence of C, Fe and Pd was approved in the catalyst (Fig. 3). In addition, the element distribution in the surface of the catalyst was studied using the meaningful pictures provided by the EDX mapping (Fig. 1S†).

##### TEM

**Fig. 4:**
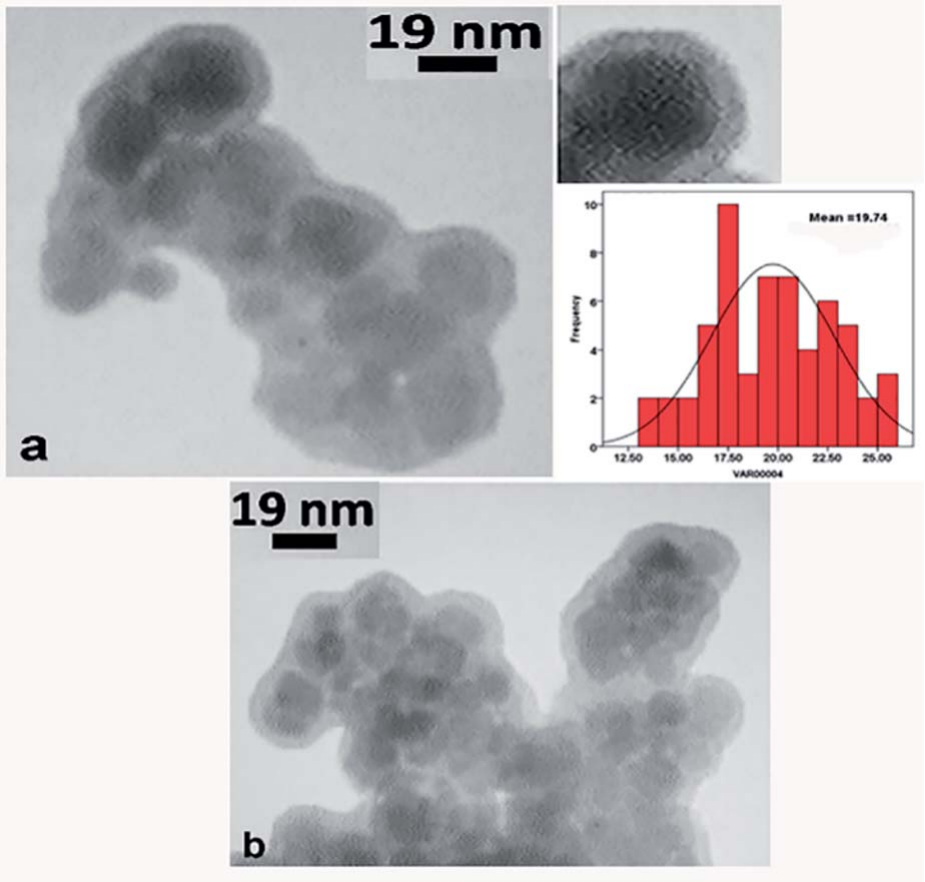
TEM images of click-MNPs/CS support (a) and Pd@click-MNPs/CS (b).

The TEM images of the click-MNPs/CS support and the catalyst Pd@click-MNPs/CS are shown in Fig. 4. The dark nano-Fe_3_O_4_ cores are surrounded by a grey polymer shell of about 3–6 nm thick and the average size of the Fe_3_O_4_ cores is about 10–20 nm (Fig. 4a). After incorporating of palladium, the Pd nanoparticles are not clearly distinguishable from Fe_3_O_4_ particles (Fig. 4b).

##### VSM

**Fig. 5:**
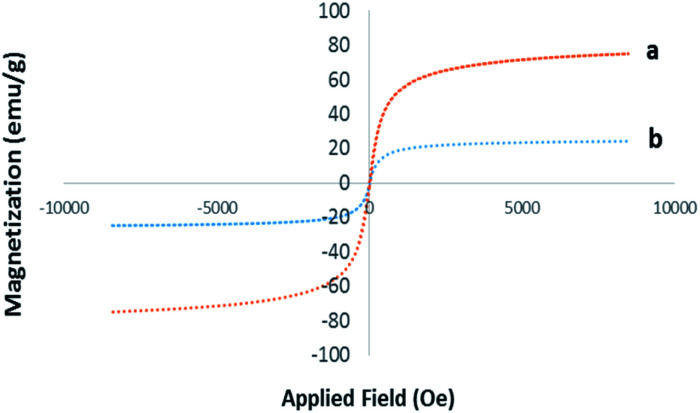
Room temperature magnetization curves of pure Fe_3_O_4_ (a) and Pd@click-MNPs/CS (b).

The magnetic properties of Pd@click-MNPs/CS were evaluated by a vibrating sample magnetometer (VSM) at room temperature. As depicted in Fig. 5, the magnetization diagram of the as-prepared material exhibits a strong magnetic property, which was also confirmed by easy and complete attraction using an external magnet.

#### 2.1.3. Behavior of 1,2,3-triazole motives in solutions

##### Interaction of 1-benzyl-4-phenyl-1*H*-1,2,3-triazole with solvents

The 1,2,3-triazole C–H hydrogen bonding and the effects of solvent on the ^1^HNMR spectrum of triazole moiety were confirmed experimentally and also with computational results. The 1-benzyl-4-phenyl-1*H*-1,2,3-triazole was prepared according to the our previous work.^46^ The ^1^HNMR spectrum of this triazole-containing compound shows the distinctive singlet of triazole C_5_–H at 8.83 ppm and 7.59 ppm in DMSO and CDCl_3_ solvents, respectively. The C_5_–H proton of 1,2,3-triazole is easily distinguished from the other protons. As shown in the HNMR spectra (Fig. 2S†), the chemical shift of the triazole C_5_–H in DMSO has been moved to downfield relative to the CDCl_3_ solvent. Based on the previous reports, the polarity and the large dipole moment (∼5 D) of 1,4-substituted-1,2,3-triazoles, that is almost aligned with the triazole C–H bond, make an electropositive C–H position which can act as an active hydrogen bond donor for anion (X^−*n*^) binding.^8^ We believe that the dielectric constant (*ε*) and Gutmann donor number (DN) of the solvents, exert a large effect on the polarization of the triazol C_5_–H bond and the acidity of the C_5_–H proton.^9,47^ The Gutmann donor number of a solvent exhibits a quantitative amount of the solvent's ability to interact with positive charge. DMSO is a very polar solvent, having a strongly polarized S–O bond, and can enter into hydrogen bonding and dipole–dipole interactions. The dielectric constant (*ε*: 46.7) and the Gutmann donor number (29.8) of DMSO is higher than that of CDCl_3_ (*ε*: 4.8, DN: 4.0). The high ability of the DMSO as a strong electron donor to interact with the acidic C_5_–H proton of triazole through hydrogen bonding (C–H⋯DMSO) is typically well associated with the downfield shift of the ^1^HNMR resonance of this CH proton, and similar correlations have been previously described by other groups for C–H⋯anion interactions.^9^

**Fig. 6:**
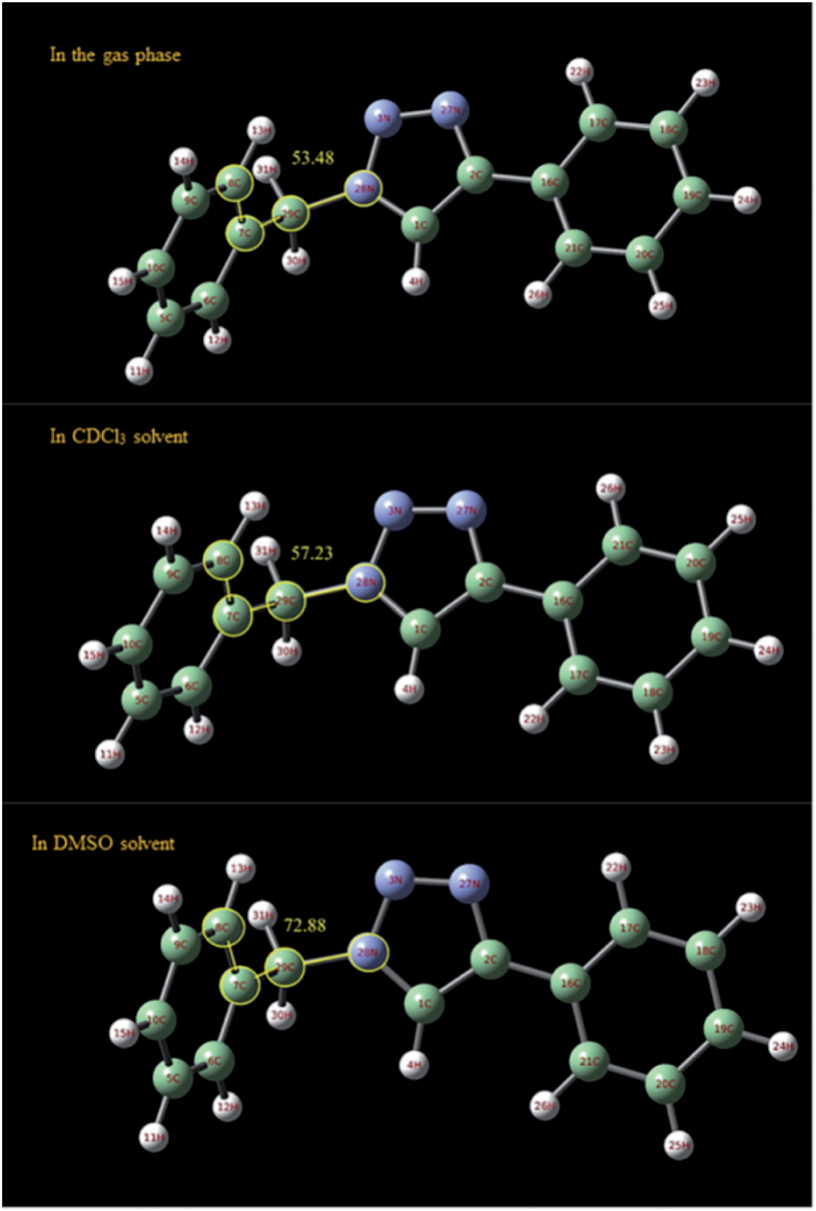
Optimized structure of 1-benzyl-4-phenyl-1*H*-1,2,3-triazole compound in the gas phase and in different solvents (DMSO and CDCl_3_) calculated at B3LYP/6-311++G(2df,p) level of theory applying SMD method. The selected dihedral angle (28N–29C–7C–8C) is 53.48, 57.23 and 72.88 in the gas phase, in the CDCl_3_ and in the DMSO solvent, respectively.

**Fig. 7:**
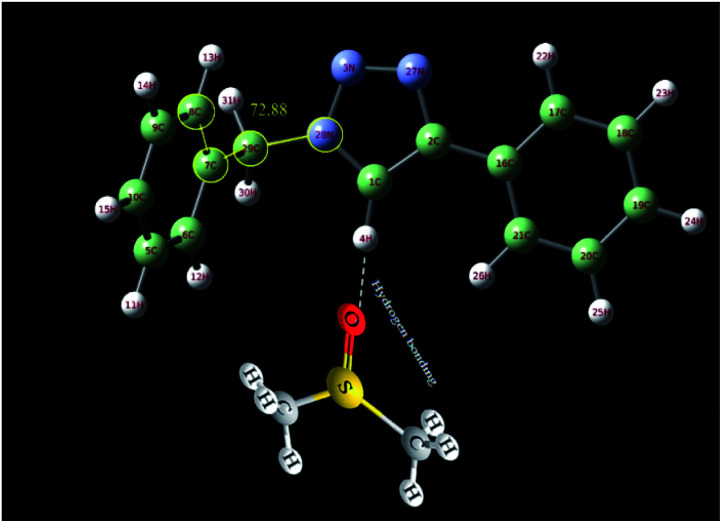
The interactions between DMSO and the triazole C–H proton.

In order to further study of the C_5_–H triazole interaction, the ^1^H NMR spectra of the 1-benzyl-4-phenyl-1*H*-1,2,3-triazole compound in the gas phase, in DMSO and in CDCl_3_ solvent have been investigated theoretically and the effect of the solvents on the most stable structure of the compound was evaluated. All the calculations have been carried out using Gaussian 09 Quantum Chemistry package.^48^ DFT methods employing Becke, 3-parameter, Lee–Yang–Parr (B3LYP) functional^49^ were used for the calculations. 6-311++G (2df, p) basis set was employed. The calculations in the presence of solvents have been done by the SMD method.^50^ At first, the structure of 1-benzyl-4-phenyl-1*H*-1,2,3-triazole compound was optimized in three different cases, in the gas phase, in DMSO and in CDCl_3_ solvent. As shown in Fig. 6, the selected dihedral angle (28N–29C–7C–8C) is 53.48, 57.23 and 72.88 in the gas phase, in the CDCl_3_ and in the DMSO solvent, respectively, which displays the influence of the solvent and its polarity on the optimized structure. The size of these dihedral angles may well confirm the existence of the interactions between DMSO and the triazole C–H proton (Fig. 7). Then, the ^1^H NMR spectra of the compound in three different cases have been calculated. The obtained results showed that the ^1^HNMR signal of triazole C–H proton was different in the different solvents with diverse polarity such as DMSO and CDCl_3_ and in the gas phase.

### 2.2. Catalytic properties of Pd-composite

In this catalyst system, the covalently-attachment of CS chains as organic section with inorganic magnetic nanoparticles *via* N-rich triazole connectors could provide a high stability and a desirable dispersion of Pd sites in the catalyst texture. Moreover, the hydrophilic nature of CS polymer provides a means of good dispersion of magnetic nanoparticles in polar medium for developing this system as a heterogeneous Pd catalyst for organic reactions in water-containing and polar reaction media.

#### 2.2.1. Carbonylation of aryl halides with formic acid

Catalytic activity of the Pd@click-MNPs/CS was evaluated in the formic acid-based carbonylation of aryl halides. The optimization process of our carbonylation methodology was initiated using 4-iodoanisole as the aryl halide substrate. The effects of the reaction conditions such as solvent, base, temperature and catalyst amount were examined on the yields of the model reaction (Table 1).

**Table 1:** Optimization of the reaction conditions for the carbonylation coupling^a^

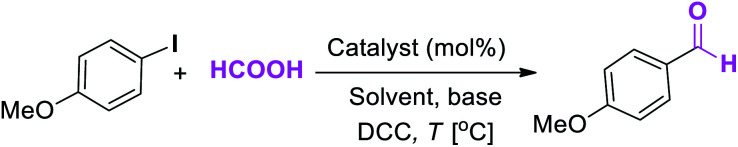						
Entry	Solvent	Base	Catalyst [mol%]	DCC	Temp. (°C)	Yield^c^ [%]
1	H_2_O/EtOH (1 : 1)	Na_3_PO_4_ (0.5 equiv.)	0.3	2 equiv.	Reflux	10
2	PEG	Na_3_PO_4_ (0.5 equiv.)	0.3	2 equiv.	105	80
3	PEG	Na_3_PO_4_ (0.5 equiv.)	0.3	2 equiv.	60	20
**4**	**PEG**	**Na**_**3**_ **PO**_**4**_ **(1 equiv.)**	**0.3**	**2 equiv.**	**105**	**85**
5	PEG	K_2_CO_3_ (0.5 equiv.)	0.3	2 equiv.	105	80
6	PEG	K_2_CO_3_ (0.5 equiv.)	0.3	—	105	25
7	PEG	K_2_CO_3_ (0.5 equiv.)	0.3	1 equiv.	105	50
8	PEG	K_2_CO_3_ (0.5 equiv.)	0.3	2 equiv.	105	60^b^
9	PEG	K_2_CO_3_ (0.5 equiv.)	0.1	2 equiv.	105	10
10	PEG	—	0.3	2 equiv.	105	60
11	PEG	K_2_CO_3_ (0.5 equiv.)	—	2 equiv.	105	—
12	PEG	DABCO (2 equiv.)	0.3	2 equiv.	105	85
13^c^	PEG	Na_3_PO_4_ (1 equiv.)	—	2 equiv.	105	—
14^d^	PEG	Na_3_PO_4_ (1 equiv.)	—	2 equiv.	105	—

a Reaction conditions:: 4-iodoanisole, HCOOH, DCC, catalyst, base, solvent (3 mL), 3 h.

b Formic acid: 4 equiv.

c Pure chitosan was used as catalyst.

d Fe_3_O_4_/chitosan composite was used as catalyst.

**Scheme 3:**
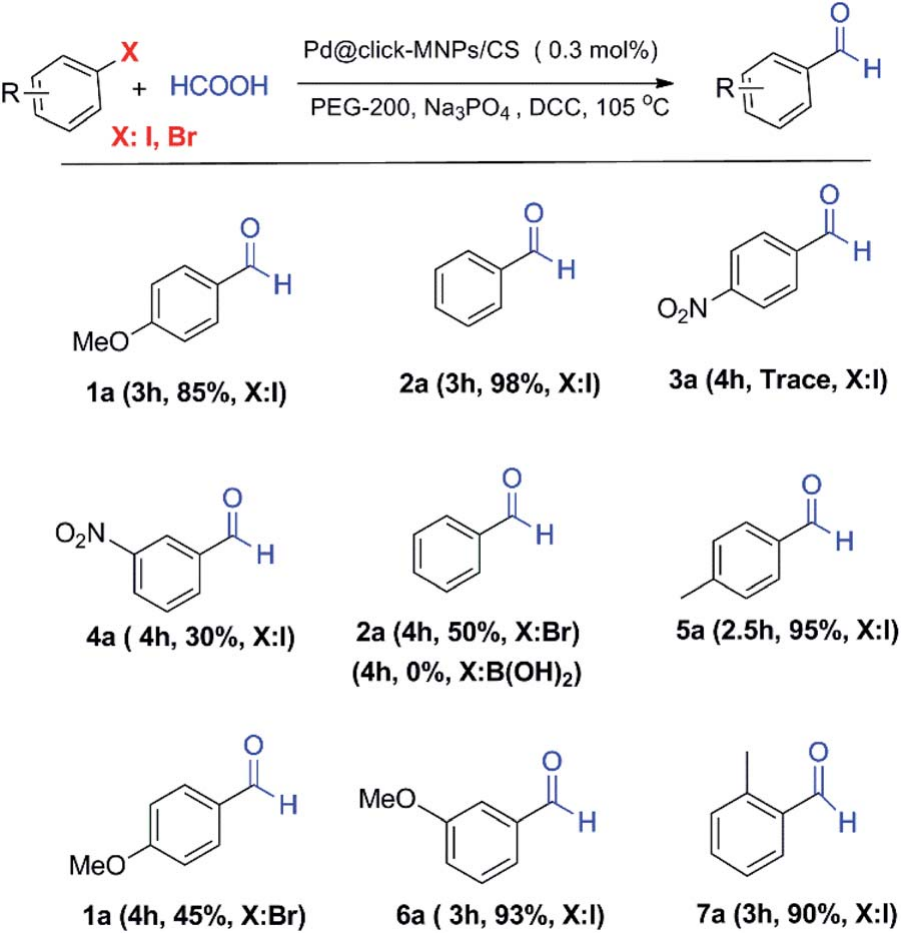
The formylation reaction using Pd@click-MNPs/CS catalyst.

In order to respect the principles of green chemistry, only eco-friendly solvents such as mixed EtOH/H_2_O (1 : 1, v/v) and polyethylene glycol (PEG-200) were tested in experiments and PEG gave the best result. The concentration of base, formic acid and DCC was also surveyed in this reaction system and found to be important. Table 1 shows a summary of the optimization experiments, in which the optimized conditions were recognized as iodobenzene (1 equiv.), HCOOH (10 equiv.), DCC (2 equiv.), catalyst loading (0.3 mol%), Na_3_PO_4_ (1 equiv.) in PEG-200 at 105 °C (Table 1, entry 4). We then explored the scope and limitations of this transformation and several aryl halides were studied (Scheme 3). The aryl iodides and bromides were active in this reaction system and aryl boronic acids were found to be inactive. The electronic effects of aryl halides functionalized with various groups such as nitro, methoxy and methyl were also examined and it was found that aryl halides substituted with electron-donating groups compared to the electron-withdrawing substituents gave better yields. The methoxy- and methyl-aryl iodides afforded the corresponding aldehydes in excellent yields; however, low yield was observed using nitro-aryl iodides as the substrate. The Scheme 3 depicts a number of aldehydes synthesized by the Pd@click-MNPs/CS catalyst.

Compared with the previous works reported by other researchers on the pd-catalyzed carbonylation reactions, the most noteworthy advantages of our formylation methodology are eco-friendly, mild, phosphine-free conditions, reusability and low Pd-loading (0.3 mol%) of catalyst, good yields and short reaction times, while in the most previous reports, about 3-5 mol% of Pd catalysts combined with the phosphine ligands promote the reaction process.^36,39,51–54^ Most of the reported formic acid-assisted formylation reactions have been performed under homogeneous catalytic conditions; meanwhile, some researchers have recently directed their studies toward the use of heterogeneous Pd-catalysts for CO gas free formylation reactions.^55,56^

**Scheme 4:**
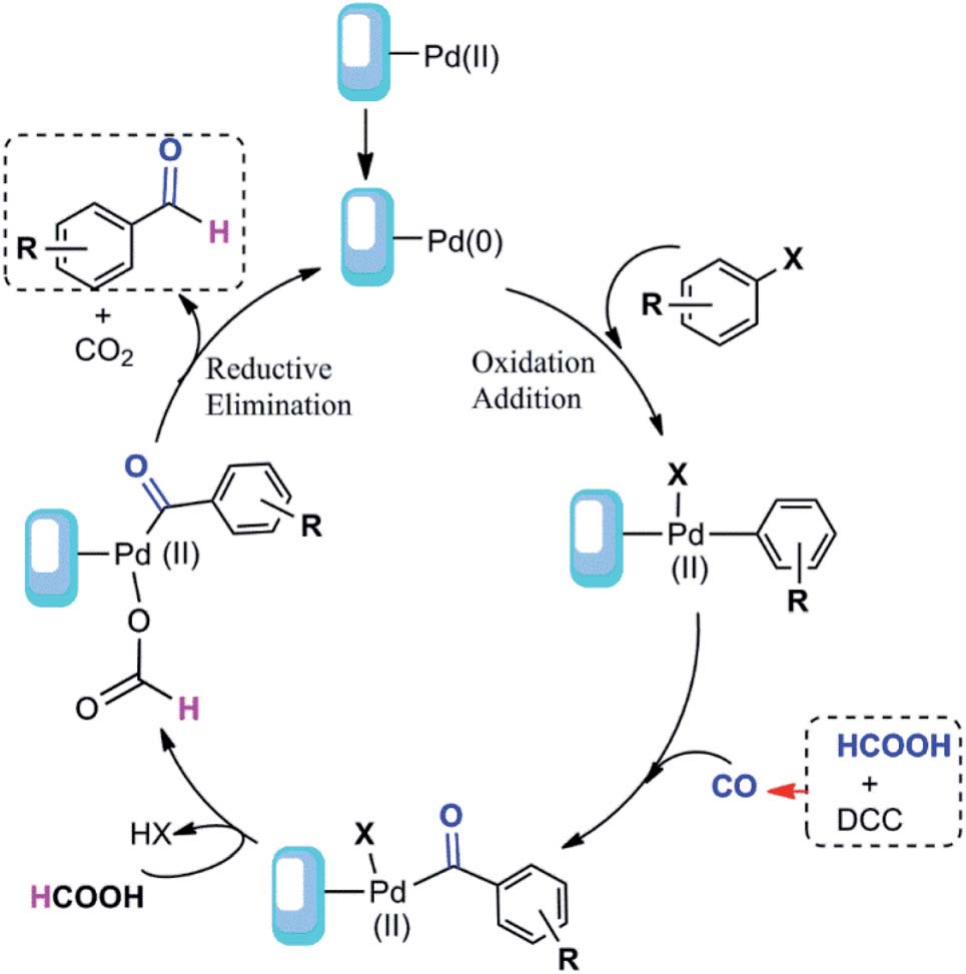
Proposed mechanism of the carbonylation reaction.

Based on our results and the other reports^52,54^ we have proposed a detailed mechanism for formic acid-assisted formylation reaction of aryl halides using Pd@click-MNPs/CS catalyst, in which the carbon monoxide molecule is formed *in situ* from the reaction of formic acid with DCC activator (Scheme 4). According to this mechanistic model, the free CO generated in the reaction media interacts with palladium-catalyst and forms an acyl-Pd intermediate in the catalytic cycle, next, followed by decarboxylation process and the reductive elimination reaction, the desired product is obtained. In this reaction system, the hydrogen source of the aldehyde products is considered to be formic acid and indeed, formic acid plays a dual role as both CO and hydrogen source to give the aldehyde groups.

The carbonyl group is one of the most common of the functional groups, and the carbonyl containing compounds are probably the most important class of organic molecules. Therefore, the construction of efficient catalytic systems for selective synthesis of these compounds will be noteworthy to follow in the near future.

#### 2.2.2. Suzuki cross-coupling reaction

Encouraged by the obtained results in the carbonylation reaction, we also investigated the potential of our Pd@click-MNPs/CS catalyst in Suzuki cross-coupling reaction. At first, the reaction conditions were optimized using the cross-coupling of 4-iodoanisole with phenylboronic acid as the model reaction. Due to environmental concerns, only EtOH and water were tested as the solvent (Table 2). Using pure water as the solvent in the presence of TBAB, moderate yield was observed (Table 2, entry 1). While, in the presence of aqueous alcohol (H_2_O/EtOH, 1 : 1 v/v) as the solvent, excellent results were achieved (Table 2, entries 2–7). The superiority of the aqueous mixed solvents could be related to the better solubility of the organic reagents and the inorganic phase in the reaction medium. Next, the influence of numerous reaction factors such as base, temperature and catalyst loading was examined (Table 2). It was observed that the base has not a significant effect on the yields in this reaction system (Table 2, entries 4–7). Based on the results, the optimized conditions were chosen as follows: EtOH/H_2_O (1 : 1, v/v) as the solvent, K_2_CO_3_ as the base with catalyst loading 0.2 mol% at room temperature (Table 2, entry 6).

**Table 2:** Optimization of the reaction conditions for the Suzuki–Miyaura coupling^a^

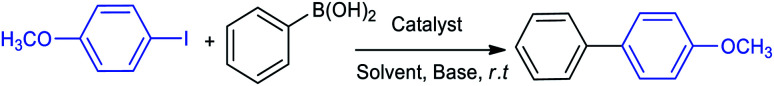				
Entry	Solvent	Base	Catalyst [mol%]	Yield^c^ [%]
1	H_2_O^b^	K_2_CO_3_	0.4	85
2	H_2_O/EtOH (1 : 1)	K_2_CO_3_	0.4	100
3	H_2_O/EtOH (1 : 1)	K_2_CO_3_	0	—
4	H_2_O/EtOH (1 : 1)	K_2_CO_3_	0.2	100
5	H_2_O/EtOH (1 : 1)	K_2_CO_3_	0.1	97
6	H_2_O/EtOH (1 : 1)	Na_3_PO_4_	0.2	100
7	H_2_O/EtOH (1 : 1)	KOH	0.2	98
8^d^	H_2_O/EtOH (1 : 1)	K_2_CO_3_	—	—
9^e^	H_2_O/EtOH (1 : 1)	K_2_CO_3_	—	—

a Reaction conditions: 4-iodoanisole (0.1 mmol), phenylboronic acid (0.12 mmol), H_2_O/EtOH (1 : 1, v/v) 2 mL, base (2 equiv.), 1 h.

b In the presence of TBAB (1 equiv.).

c GC yield.

d Pure chitosan was used as catalyst.

e Fe_3_O_4_/chitosan composite was used as catalyst.

After optimization, we examined the reactivity of various types of aryl halides in this catalytic reaction system and it was found that the different biaryl derivatives were successfully synthesized by Pd@click-MNPs/CS catalyst using the Suzuki cross-coupling process (Table 3).

**Table 3:** Scope of aryl halides in Suzuki–Miyaura coupling reaction with phenylboronic acid a

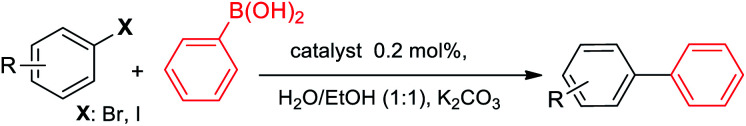				
Entry	R (X)	*T* [°C]	*t* [h]	Yield^b^ [%]
1	4-OCH_3_ (I)	r.t.	1	100
2	4-NO_2_ (I)	r.t.	1	100
3	4-CH_3_ (I)	r.t.	2	96
4	4-COCH_3_ (I)	r.t.	2	100
5	3-NO_2_ (I)	r.t.	1	100
6	H (I)	r.t.	1	95
7	4-COCH_3_ (Br)	r.t.	2	95
8	3-COCH_3_ (Br)	80	20	50
9	4-OCH_3_ (Br)	r.t.	1	100
10	4-COH (Br)	r.t.	2	95
11	4-CN (Br)	r.t.	1	100
12	4-Cl (Br)	r.t.	1	100
13	2-Cl (Br)	r.t.	3	95
14	4-NO_2_ (Br)	r.t.	20	25
15	4-NO_2_ (Br)	70	1	95
16	2-NO_2_ (Br)	70	20	—
17	H (Cl)	r.t.	3	—
18	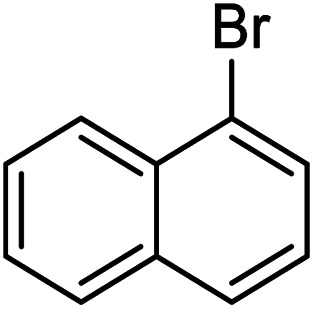	r.t.	6	90

a Reaction conditions: aryl halide (1 mmol), Phenylboronic acid (1.2 mmol), K_2_CO_3_ (2 mmol), solvent 3 mL.

b GC yield.

The electronic effects on the reaction times and yields were evaluated and electron-rich as well as electron-poor aryl iodides proceeded excellently to give the biaryl products. However, the reaction of nitro-aryl bromides afforded low yields at room temperature (Table 3 entry 14), while in contrast, methoxy-aryl bromide required shorter reaction times and gave brilliant yields (Table 3 entry 9). In the case of 4-nitrobromobenzene, when the reaction performed at room temperature, the corresponding coupled product was achieved in 25% of yield after 20 h, however, the yield of the product increased to 95% when the reaction was conducted at 70 °C for 1 h. Various functional groups such as cyano, methoxy, halogen, and carbonyl substituted on the aryl halides were compatible with this pd-catalyst system. Aryl chlorides were inactive in this system. The steric effects was evaluated and it was observed that an increasing hindrance in the vicinity of the leaving group using 2-bromonitrobenzene (Table 3, entry 16) led to a fall in the yield, however, the reaction by 2-bromochlorobenzene required long times, giving excellent conversion (Table 3, entry 13).

**Scheme 5:**
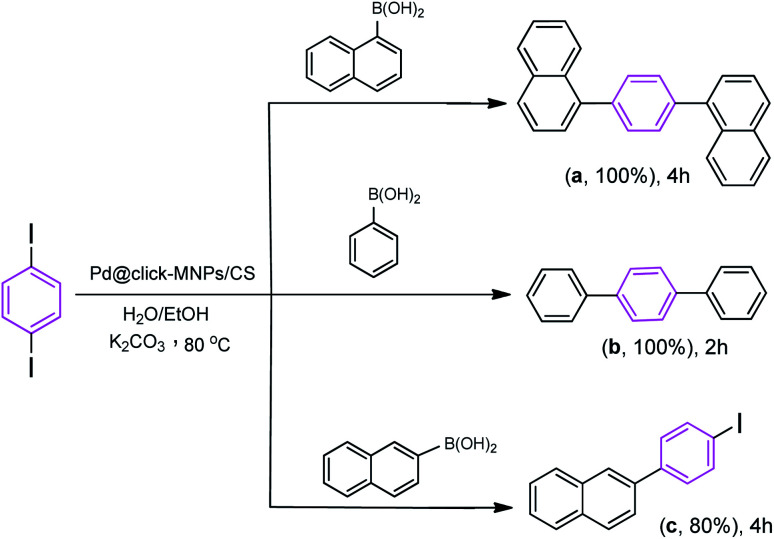
Reaction conditions: 1,4-diiodobenzene (1 equiv.), arylboronic acid (2.2 equiv.), catalyst (0.4 mol%), K_2_CO_3_ (4 equiv.), H_2_O/EtOH (1 : 1, v/v), 80 °C.

We also studied the efficiency of catalyst using 1,4-diiodobenzene as the substrate with various boronic acids (Scheme 5). It was found that the treatment of 1,4-diiodobenzene (1 equiv.) with phenylboronic acid (2 equiv.) in the presence of K_2_CO_3_ as the base (4 equiv.), H_2_O/EtOH (1 : 1, v/v) as the solvent with catalyst loading 0.4 mol% at room temperature affords the coupled product 1,1':4′,1′′-terphenyl (b) in 75% yield after 2 h, however, under the same reaction conditions, the use of reaction temperature (80 °C) increases the yield of the corresponding coupled product up to 100%. Under these conditions, the reaction of 1,4-diiodobenzene substrate with naphthalene-1-boronic acid and naphthalene-2-boronic acid was also investigated. As shown in Scheme 5, both iodide groups in 1,4-diiodobenzene were completely reacted with naphthalene-1-boronic acid and the corresponding coupled product 1,4-di(naphthalen-1-yl)benzene (a) was formed in excellent yield. However, in the reaction of 1,4-diiodobenzene with naphthalene-2-boronic acid, the Suzuki product 2-(4-iodophenyl)naphthalene (c) was formed as the main product.

**Scheme 6:**
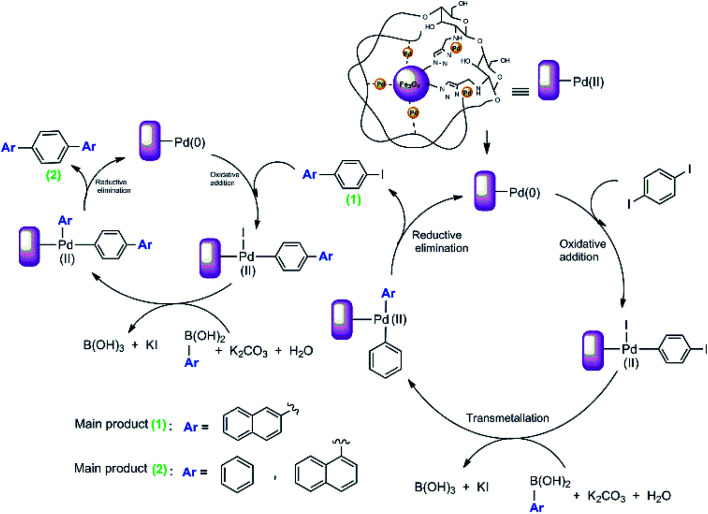
The Suzuki reaction mechanism of 1,4-diiodobenzene.

We proposed a possible mechanism for the Suzuki–Miyaura coupling of 1,4-diiodobenzene with arylboronic acids using Pd@click-MNPs/CS as the catalyst, which is in consisted with previous reports.^57^ As shown in Scheme 6, a Pd(0)/Pd(ii) catalytic cycle *via* an oxidative addition/reductive elimination mechanistic pathway has been reported, in which a complex Pd(ii) intermediate undergoes reductive elimination to expel the coupled products.

The present catalyst system was compared with published reaction conditions reported by other groups for the Suzuki coupling of 4-bromoanisole with phenylboronic acid, and the results are summarized in Table 4. The present catalyst can be one of the best catalysts in terms of low reaction time, lower temperature, green media, easy separation and efficient recycling.

**Table 4:** Comparison of the catalytic activity with literature examples for the Suzuki reaction between 4-bromoanisole and phenylboronic acid

Entry	Catalyst ^Ref^	Reaction conditions	Yield (%)
1	Pd_74_Cu_73_ DENs^58^	Catalyst (1 mol%), EtOH/H_2_O (3 : 1), K_2_CO_3_, MW 50 °C, 0.75 h	99.7
2	HEC-NHC-Pd^59^	Catalyst (0.4 mol%), EtOH/H_2_O (3 : 2), K_2_CO_3_, 60 °C, 2 h	84
3	PET@IL/Pd^60^	Catalyst (0.1 mol%), H_2_O, K_2_CO_3_, 55 °C, 45 min	80
4	MOP-BPY(Pd)^61^	Catalyst (0.1 mol%), MeOH/H_2_O (1 : 1), Na_2_CO_3,_ 80 °C, 12 h	91.3
5	Present work	Catalyst (0.2 mol%), EtOH/H_2_O (1 : 1), K_2_CO_3_, rt, 1 h	100

### 2.3 Efficiency and stability of Pd-composite

**Scheme 7:**
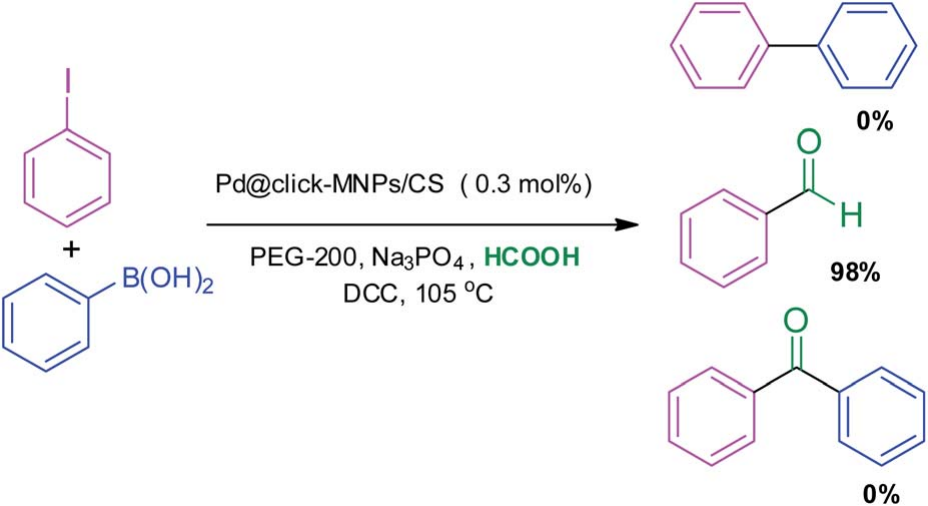
Reaction selectivity of Pd@click-MNPs/CS for the Suzuki and carbonylation reaction.

To obtain more information about the selectivity and reactivity of Pd@click-MNPs/CS catalyst, we designed a reaction mixture containing the Suzuki and carbonylation reaction experimental conditions (Scheme 7). It was found that by employing iodobenzene (1 equiv.), phenylboronic acid (1.2 equiv.), Na_3_PO_4_ (2 equiv.), catalyst (0.3 mol%), DCC (2 equiv.) and HCOOH (10 equiv.) in the PEG-200 solvent at 105 °C, the benzaldehyde was obtained as the only reaction product in 98% yield after 3 h.

**Fig. 8:**
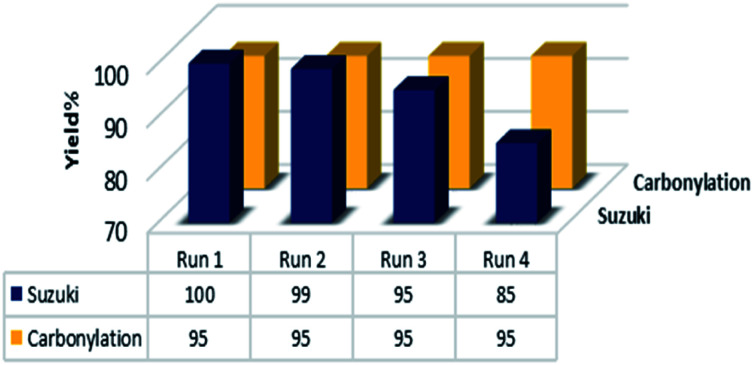
The reusability results of the catalyst. Suzuki: 4-iodoanisole, carbonylation 4-iodotoluene as the substrates under the optimized conditions.

The recyclability of heterogeneous catalyst is a key factor that is directly related to the catalyst stability and is a very essential topic from both the economic and environment points of view, especially for costly pd-based catalysts. Therefore, we carried out further investigations about the reusability and recovery of the Pd@click-MNPs/CS catalyst using the carbonylation and Suzuki cross-coupling reaction. After completing each reaction, the magnetic catalyst could be efficiently separate from the reaction media by an external magnet and reused for the next run. As demonstrated in Fig. 8, the catalyst could be recycled for at least four times without significant decrease in the catalytic activity.

**Fig. 9:**
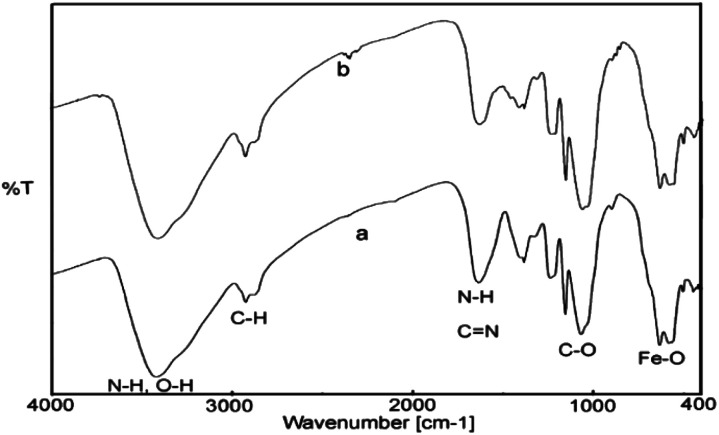
The FT-IR spectra of fresh (a) and recovered (b) catalyst.

The FT-IR spectra of the fresh and recovered catalyst from Suzuki reaction have been shown in Fig. 9. As can be seen, the recycled catalyst exhibited characteristic peaks of the chitosan (N–H, O–H, C–H, C–O) and Fe_3_O_4_ (Fe–O–Fe) similar to those of the fresh one, which indicate the stability of catalyst structures during the reaction process in aqueous media.

## 3. Conclusions

The present work is a study on the potential of 1,2,3-triazole frameworks in C–H⋯X hydrogen bonding and metal-based catalyst design. The triazoles prepared from azides and alkynes are compounds beyond the sum of their clicking building blocks. These N-heterocyclic frameworks offer the interactions with both metal cations and anion species. The environmentally friendly nanocatalyst Pd@click-MNPs/CS exhibited a good catalytic activity in CO gas-free carbonylation reaction of aryl halides and HCOOH to prepare a diversity of aromatic aldehydes in moderate to excellent yields. This heterogeneous nanocatalyst was also evaluated in Suzuki cross-coupling reaction of diverse aryl halides and phenylboronic acid in aqueous solvent at room temperature. The utilize of triazole entities as the C–H hydrogen bond donor, stable linker and powerful N-donor ligand, especially for the surface modification, covalently attachments and the catalyst immobilization is noteworthy to follow in the future researches due to effective and facile construction of these frameworks.

## 4. Experimental

### 4.1. Synthesis of Fe_3_O_4_ nanoparticles

A solution of Iron(iii) chloride and Iron(ii) chloride (1 : 2 molar ratio) was prepared in a three-necked round bottom flask using deionized (DI) water under nitrogen with vigorous stirring at 85 °C.^62^ Then, ammonia solution was added gradually into the above mixture while the reaction mixture was stirred strongly until pH reached about 9. After 2 h stirring at 85 °C, a black precipitate (Fe_3_O_4_) was magnetically collected, washed with distilled water and ethanol and dried.

### 4.2. Synthesis of azide-decorated Fe_3_O_4_ nanoparticles

At first, the 3-azidopropyltrimethoxysilane was prepared using 3-chloropropyltrimethoxysilane.^19^ 3-Chloropropyltrimethoxysilane (0.92 mL, 5 mmol), tetrabutylammonium bromide (TBAB, 0.32 g, 1 mmol), NaN_3_ (0.46 g, 7 mmol) and dry acetonitrile (30.0 mL) were mixed under nitrogen atmosphere. The resulting mixture was refluxed for 24 h. After completion and cooling, the acetonitrile was evaporated using rotary. The residue was then treated with Et_2_O (20 mL), filtered and washed with Et_2_O. The etheric phase was evaporated to obtain 3-azidopropyltrimethoxysilane, and the product was mixed with 50 mL ethanol for the next step reaction with Fe_3_O_4_ nanoparticles.

The fresh prepared Fe_3_O_4_ particles (1 g) were ultrasonically suspended in ethanol and then the solution of 3-azidopropyltrimethoxysilane in ethanol was added. The mixture was heated at 40 °C under nitrogen for 18 h. Then, the obtained azide-functionalized magnetic particles were magnetically separated and washed thoroughly with ethanol and dried under vacuum. The production of 3-azidopropyltrimethoxysilane and the successful grafting of azide groups on the magnetic particles surface were approved using FT-IR analysis.

### 4.3. Synthesis of alkyn-functionalized chitosan

Propargyl bromide (8 mmol) was added dropwise, while stirring to a mixture of DMF (10 mL) and 0.3 g chitosan (MW: 190 000–310 000, DD: 84%) in a round-bottom flask. The mixture was stirred at room temperature for 2 h and heated at 60 °C for 20 h. Next, the reaction mixture was cooled to room temperature and added gradually to a beaker containing distilled water to precipitate the alkynylated chitosan. The resulting solid was collected by centrifugation, washed thoroughly with acetone several times and dried. FT-IR (KBr, cm^−1^): 3432 (OH), 2926–2850 (aliphatic C–H), 2100 (C≡C), 1072 (C–O).

### 4.4. Synthesis of click-MNPs/CS

Azide-functionalized Fe_3_O_4_ particles (0.1 g) and alkynylated chitosan (0.2 g) were mixed in 15 mL H_2_O/DMF (1 : 1), and then Cu_2_O (0.015 g) was added as the catalyst. The reaction mixture was stirred vigorously and heated in an oil bath at 60 °C for 3 d. After completion, the solid particles were magnetically separated, washed several times with MeOH, H_2_O and ammonia solution (to remove copper impurities) and then, dried under vacuum.

### 4.5. Synthesis of Pd@click-MNPs/CS

The click-MNPs/CS (0.1 g) was sonicated for 5 min and mixed with palladium acetate (0.008 g) in dry toluene (10 mL). The reaction mixture was stirred at room temperature for 2 days. Then, the magnetic phase was separated by a permanent magnet, washed with toluene and acetone several times and dried at room temperature.

### 4.6. General procedure for the carbonylation reaction

Aryl halide (1 mmol), Na_3_PO_4_ (1 mmol), catalyst (0.3 mol%), DCC (2.0 mmol) and PEG-200 (3.0 mL) were mixed in a round-bottom flask. Then, formic acid (10 mmol) was added to the reaction mixture, immediately sealed, and the mixture was heated in an oil bath at 105 °C. After the completion of the reaction process and cooling, the CH_2_Cl_2_ was added to the reaction mixture and the catalyst was separated by a permanent magnet, washed with CH_2_Cl_2_/acetone and then reused for recycling experiments. The crude product was purified by silica gel-column chromatography to obtain the corresponding aldehyde product.

### 4.7. General procedure for Suzuki–Miyaura coupling reaction

Aryl halide (1 mmol), K_2_CO_3_ (2.0 mmol), phenylboronic acid (1.2 mmol), catalyst (0.2 mol%) and 3 mL H_2_O/EtOH (1 : 1) were transferred into a round-bottom flask. The reaction mixture stirred at room temperature and monitored by gas chromatography (GC). After completion, the reaction mixture was diluted with EtOH and the catalyst was magnetically recovered by a permanent magnet, washed with EtOH and then reused. The corresponding biaryl product was extracted with EtOAc several times and the organic phase was collected, dried over CaCl_2_, filtered and concentrated. The purified product was obtained by recrystallization or column chromatography (EtOAc : *n*-hexane) on silica gel, and characterized.

## Conflicts of interest

All authors declare that they have no conflict of interest associated with this publication.

## Supplementary Material

RA-011-D1RA03356E-s001
