# Supplementary material for: 1,2,3-Triazole framework: a strategic structure for C–H⋯X hydrogen bonding and practical design of an effective Pd-catalyst for carbonylation and carbon–carbon bond formation
Source: RSC Adv. 2021 Jun 11;11(34):20812–23. doi: 10.1039/d1ra03356e (PMC9034039; doi:10.1039/d1ra03356e)
Supplement: RA-011-D1RA03356E-s001 [file RA-011-D1RA03356E-s001.pdf]

Electronic Supplementary Material (ESI) for RSC Advances.
This journal is © The Royal Society of Chemistry 2021

Supporting Information

1,2,3-Triazole Framework: A Strategic Structure for C-H•••X Hydrogen Bonding and Practical Design of an Effective Pd-Catalyst for Carbonylation and Carbon-Carbon Bond Formation

Fatemeh Mohammadsaleh*[a], Maryam Dehdashti Jahromi[b], Abdol Reza Hajipour[c, d], Seyed Mostafa Hosseini[c] and Khodabakhsh Niknam*[a]

[a] aDepartment of Chemistry, Faculty of Nano and Bio Science and Technology, Persian Gulf University, Bushehr, Iran. E-mail address: f.mohammadsaleh@gmail.com, niknam@pgu.ac.ir

[b]Faculty of engineering, Jahrom University, Jahrom, Iran

[c]Department of Chemistry, Pharmaceutical and Organic Synthesis Research Laboratory, Isfahan University of Technology, Isfahan 84156, Iran

[d]Department of Pharmacology,University of Wisconsin, Medical School, 1300 University Avenue, Madison, 53706-1532 WI, USA

Table of Contents

1. **General Remarks**
2. **Elemental EDX mapping of the catalyst Pd@click-MNPs/CS**
3. **Characteristic data of the products**

1) General Remarks

All reagents were purchased from commercial suppliers (Merck and Aldrich) and used without further purification. The FT-IR spectra of products were measured as KBr disc by Jasco-680 FTIR spectrophotometer. The X-ray diffraction (XRD) patterns were recorded by using a Philips Xpert MPD diffractometer with Cu Ka radiation (l = 0.15418 nm). Transmission Electron Microscopy (TEM) was carried out on Philips CM120 microscope 100 KV. Scanning electron microscopy (FE-SEM) images were obtained using a Hitachi S-4160 instrument. Analysis of Pd content was measured by inductively coupled plasma spectroscopy (ICP) using Perkin Elmer Optima 7300 DV .Melting points were determined by a Gallenkamp melting apparatus. Gas chromatography (GC) analyses were performed on a BEIFIN 3420 gas chromatograph equipped with a Varian CP SIL 5CB column: 30 m, 0.32 mm, 0.25 mm for consideration of reactions conversions and yields. The ^{1}H and ^{13}C NMR spectra were recorded on a Bruker AVANCE spectrometer at 400, 300 and 100 MHz using TMS as an internal standard in $CDCl_3$ and DMSO at RT.

2) Elemental EDX mapping of the catalyst Pd@click-MNPs/CS (Figure 1S.)

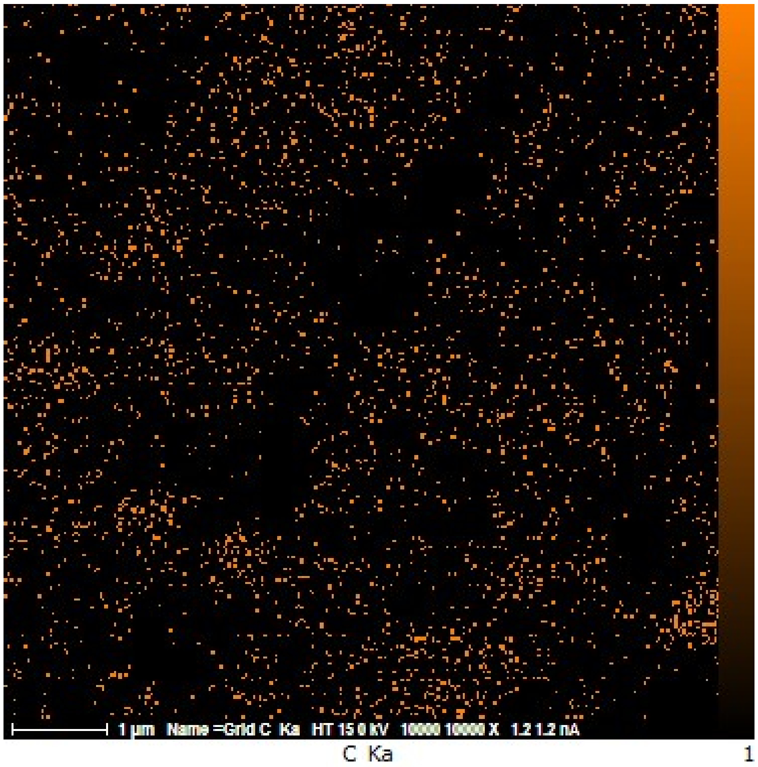

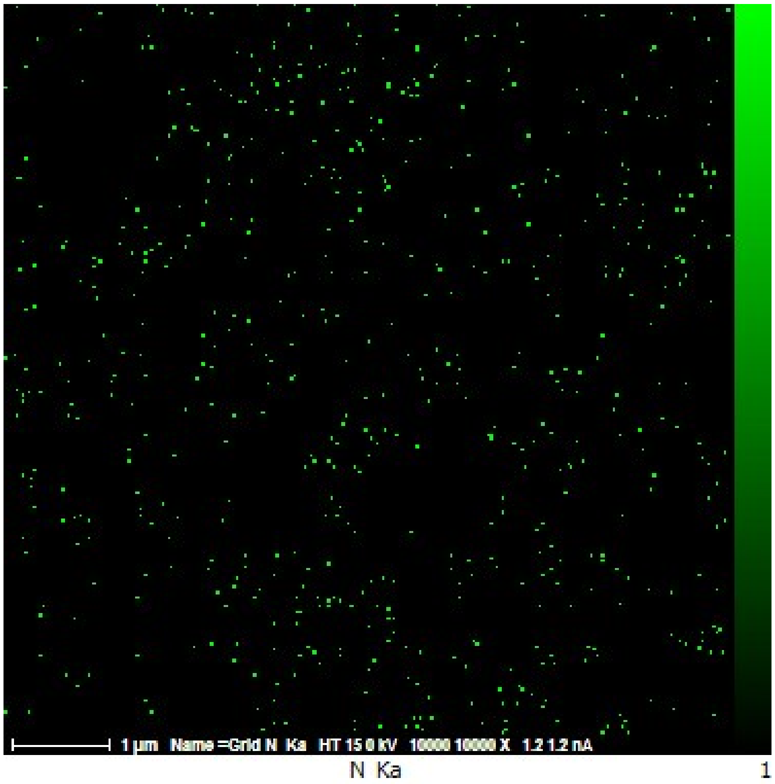
1 µm Name =Grid N Ka HT 15.0 kV 10000 10000 X 1.2 1.2 nA
N Ka
1

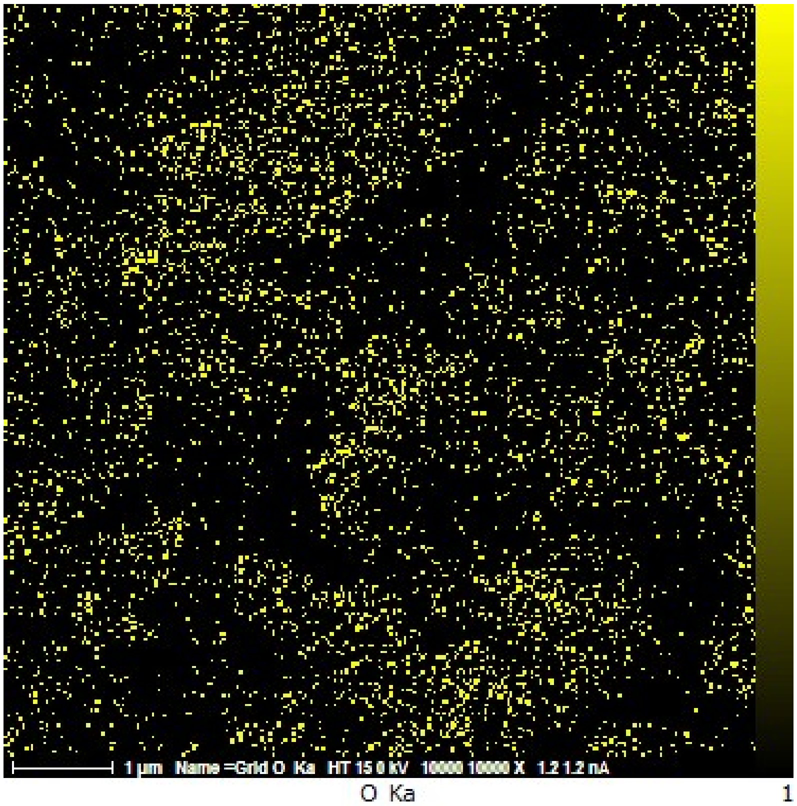
1 µm Name =Grid O Ka HT 15.0 kV 10000 10000 X 1.2 1.2 nA
O Ka
1

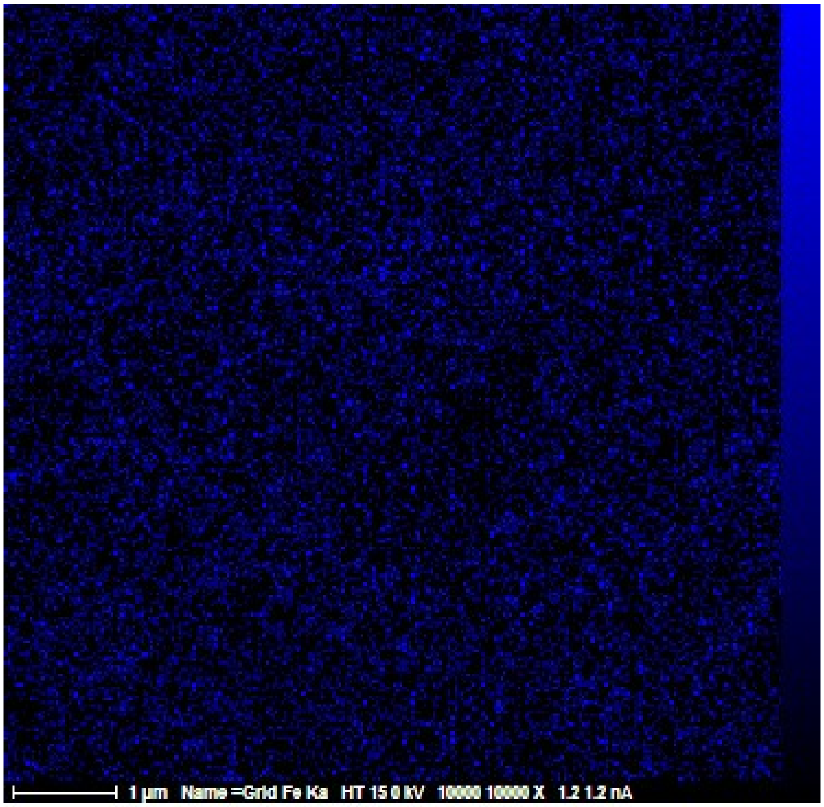
1 µm Name =Grid Fe Ka HT 15 0 kV 10000 10000 X 1.2 1.2 nA

Fe Ka 2

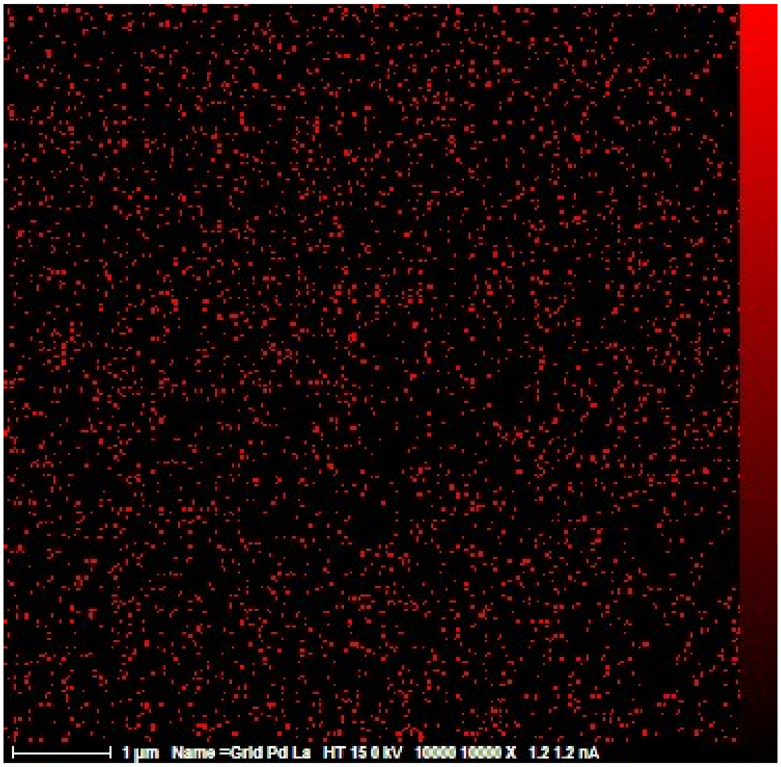
1 µm Name =Grid Pd La HT 15 0 kV 10000 10000 X 1.2 1.2 nA

Pd La 1

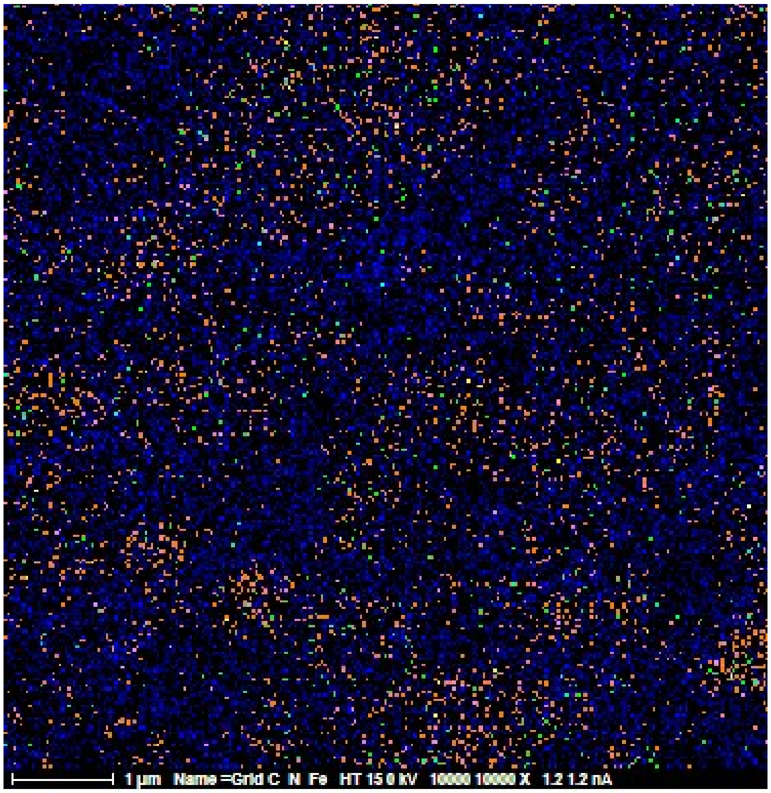
1 µm Name =Grid C N Fe HT 15 0 kV 10000 10000 X 1.2 1.2 nA
C N Fe

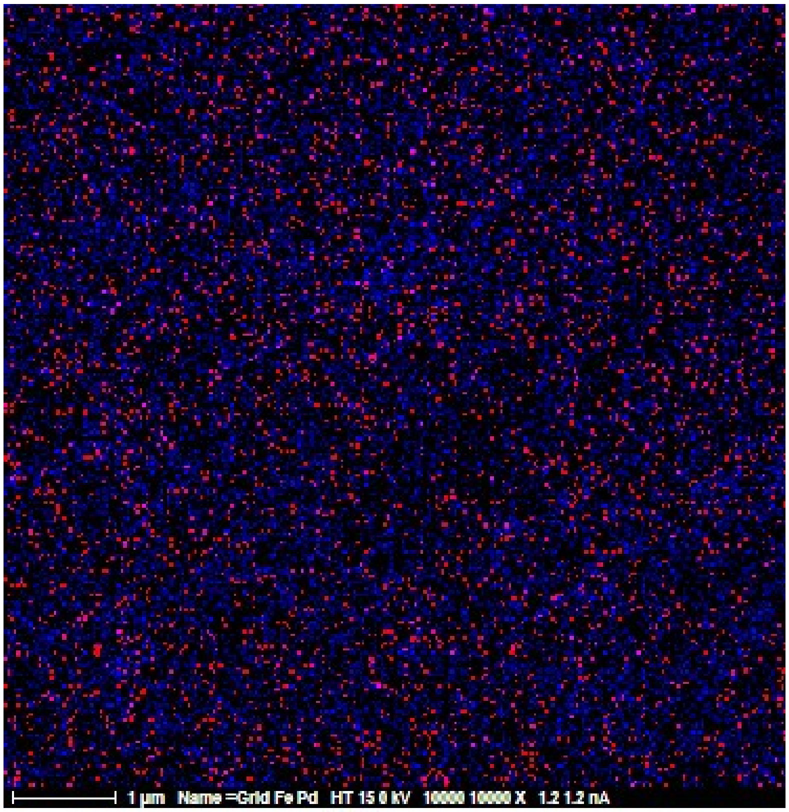
1 µm Name =Grid Fe Pd HT 15 0 kV 10000 10000 X 1.2 1.2 nA
Fe Pd

3) Characteristic spectra of the products

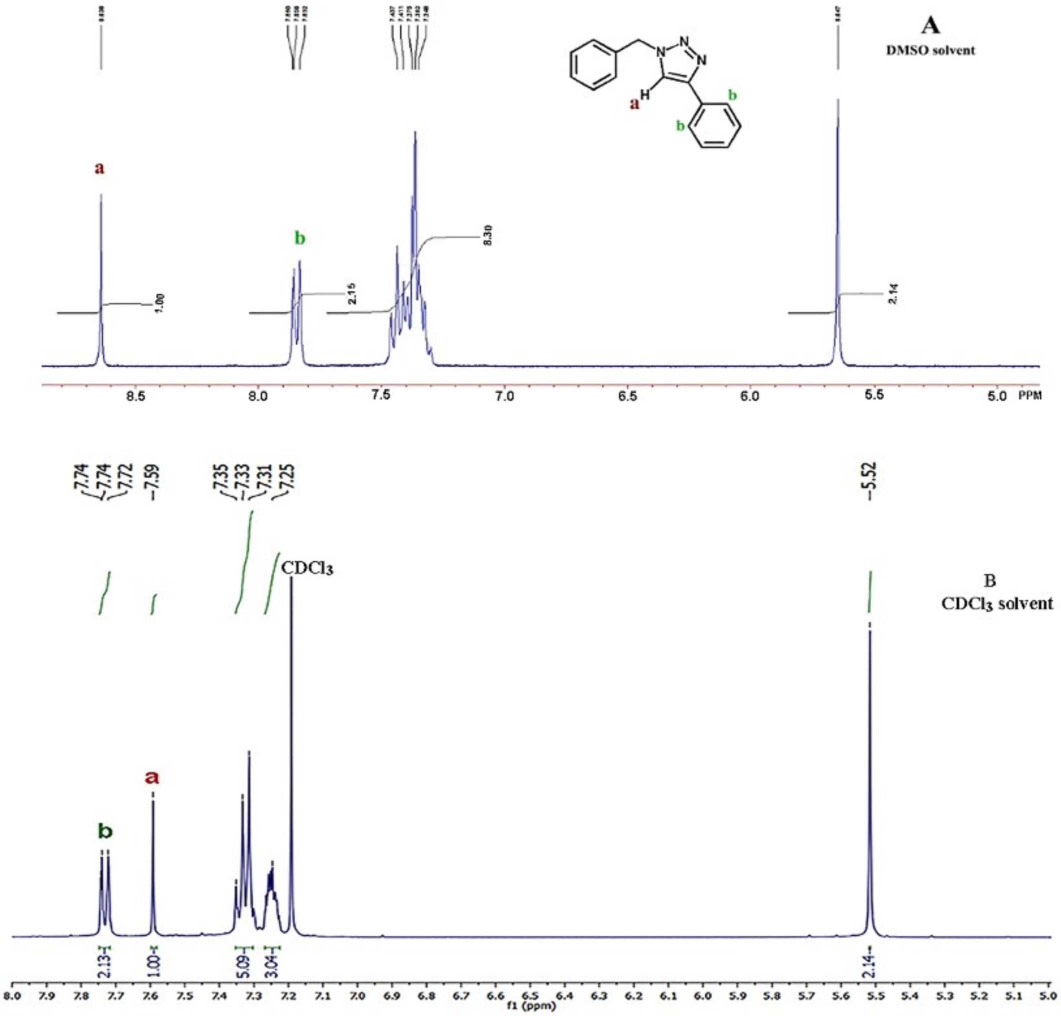

Figure 2S. ^{1}HNMR spectra of 1-benzyl-4-phenyl-1-*H*-1,2,3-triazole in DMSO and $CDCl_3$ solvent

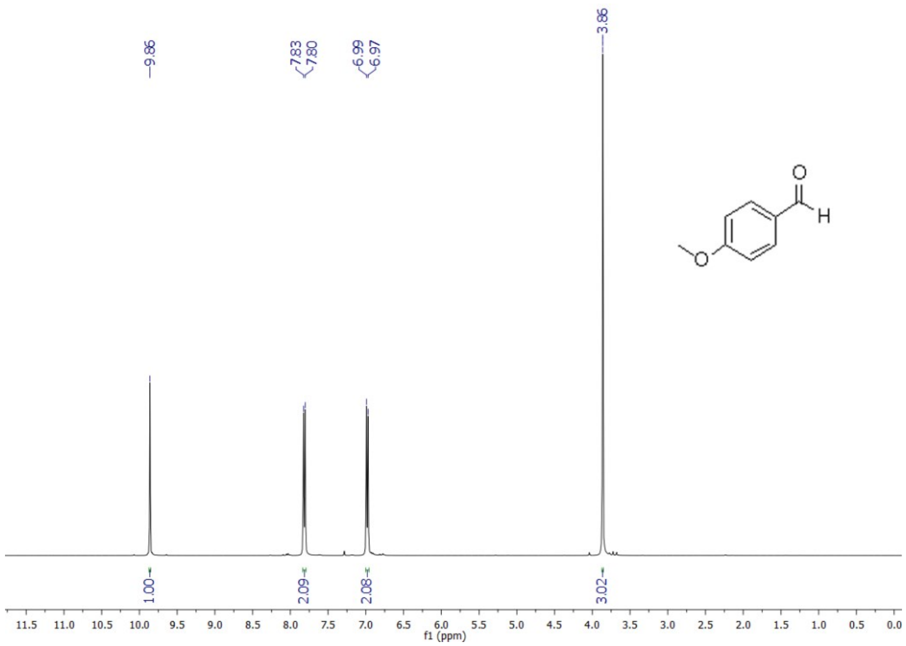
9.86
7.83
7.80
6.99
6.97
3.86
1.00
2.09
2.08
3.02
11.5 11.0 10.5 10.0 9.5 9.0 8.5 8.0 7.5 7.0 6.5 6.0 5.5 5.0 4.5 4.0 3.5 3.0 2.5 2.0 1.5 1.0 0.5 0.0
f1 (ppm)
O
H
O

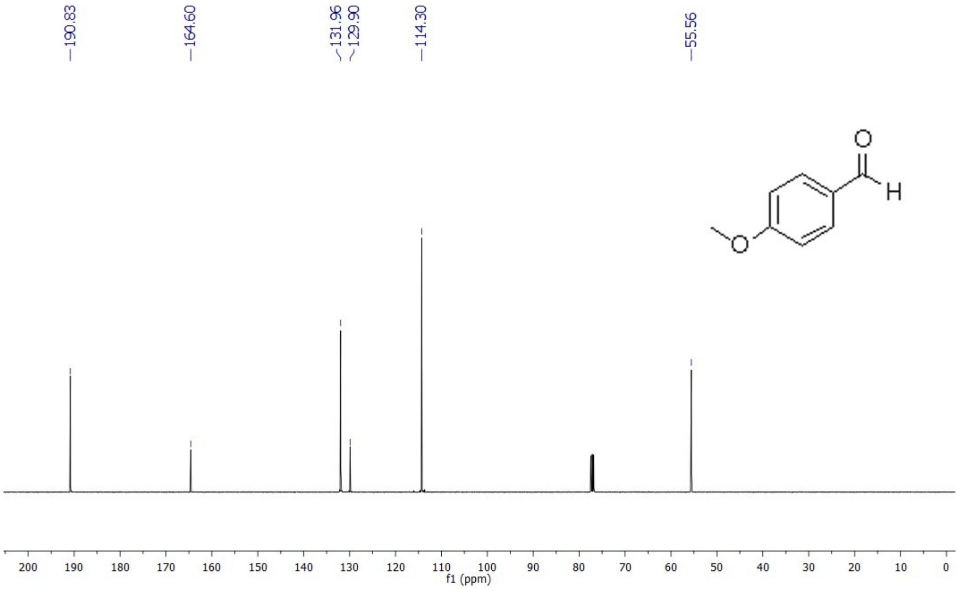
190.83
164.60
131.96
129.90
114.30
55.56
200 190 180 170 160 150 140 130 120 110 100 90 80 70 60 50 40 30 20 10 0
f1 (ppm)
O
H
O

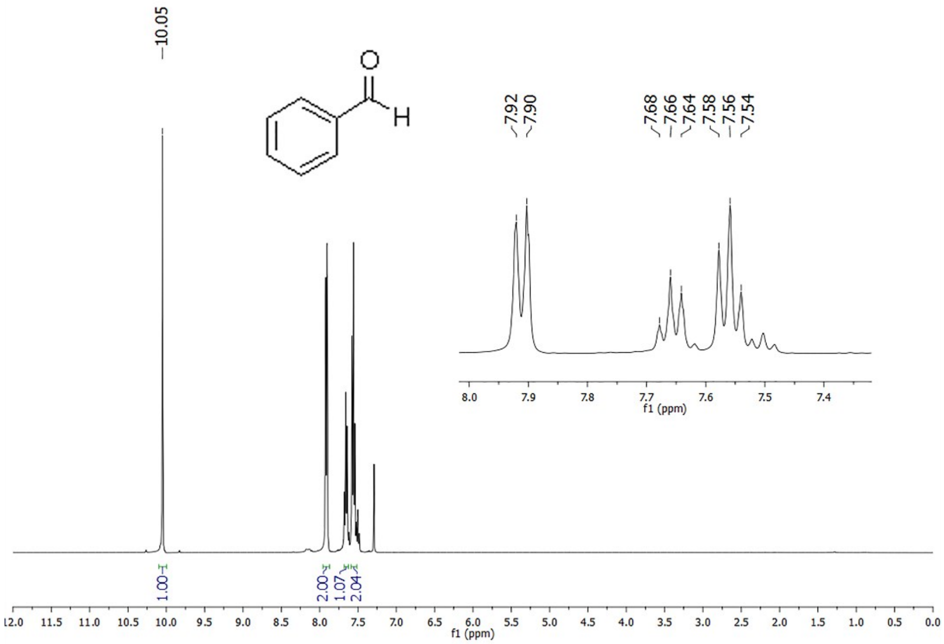

10.05
7.92
7.90
7.68
7.66
7.64
7.58
7.56
7.54
1.00
2.00
1.07
2.04
f1 (ppm)

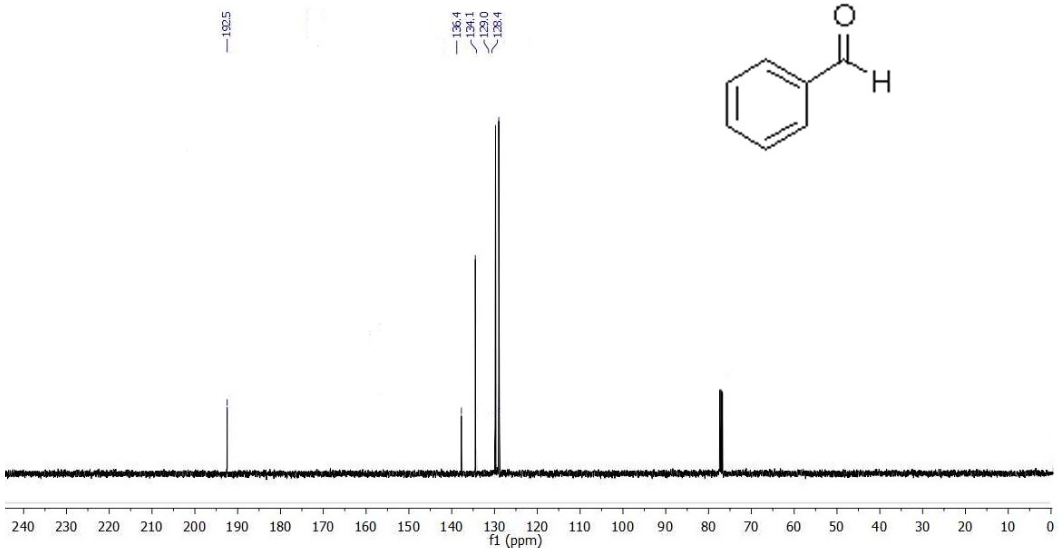

192.5
136.4
134.1
129.0
128.4
f1 (ppm)

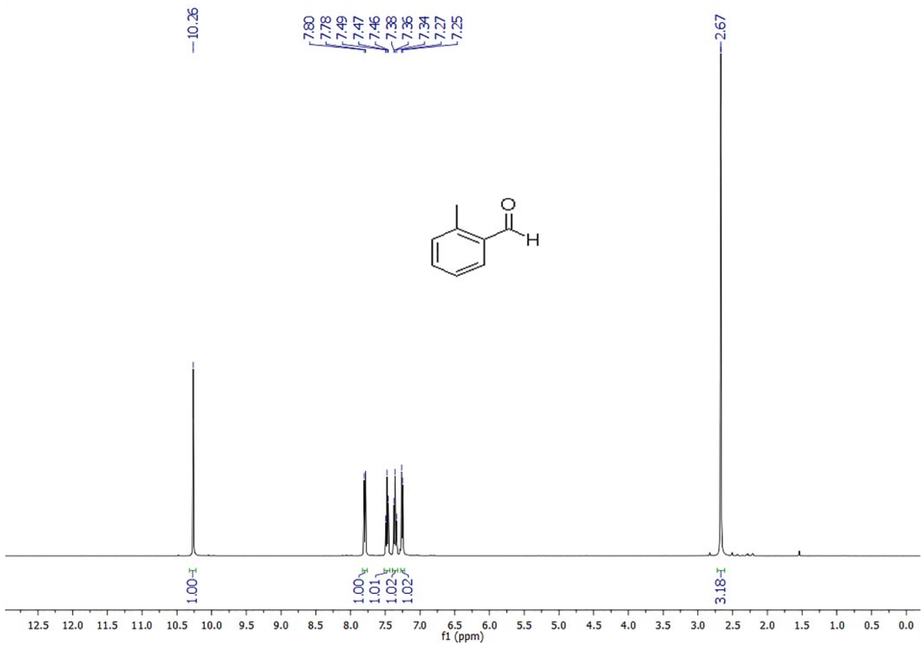

10.26
7.80
7.78
7.49
7.47
7.46
7.38
7.36
7.34
7.27
7.25
2.67
H
1.00
1.00
1.01
1.02
1.02
3.18
12.5 12.0 11.5 11.0 10.5 10.0 9.5 9.0 8.5 8.0 7.5 7.0 6.5 6.0 5.5 5.0 4.5 4.0 3.5 3.0 2.5 2.0 1.5 1.0 0.5 0.0
f1 (ppm)

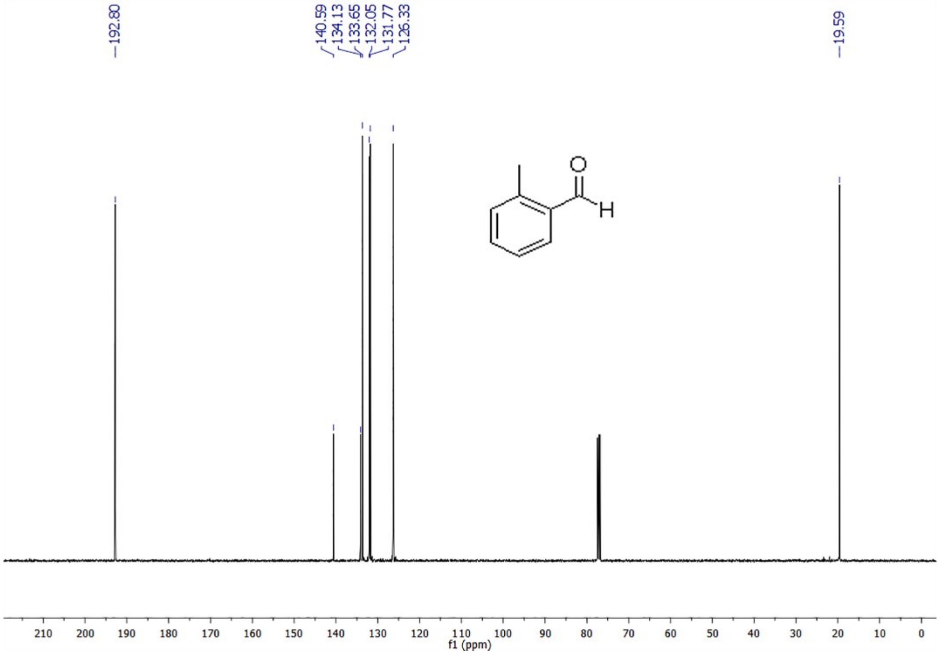

192.80
140.59
134.13
133.65
132.05
131.77
126.33
19.59
H
210 200 190 180 170 160 150 140 130 120 110 100 90 80 70 60 50 40 30 20 10 0
f1 (ppm)

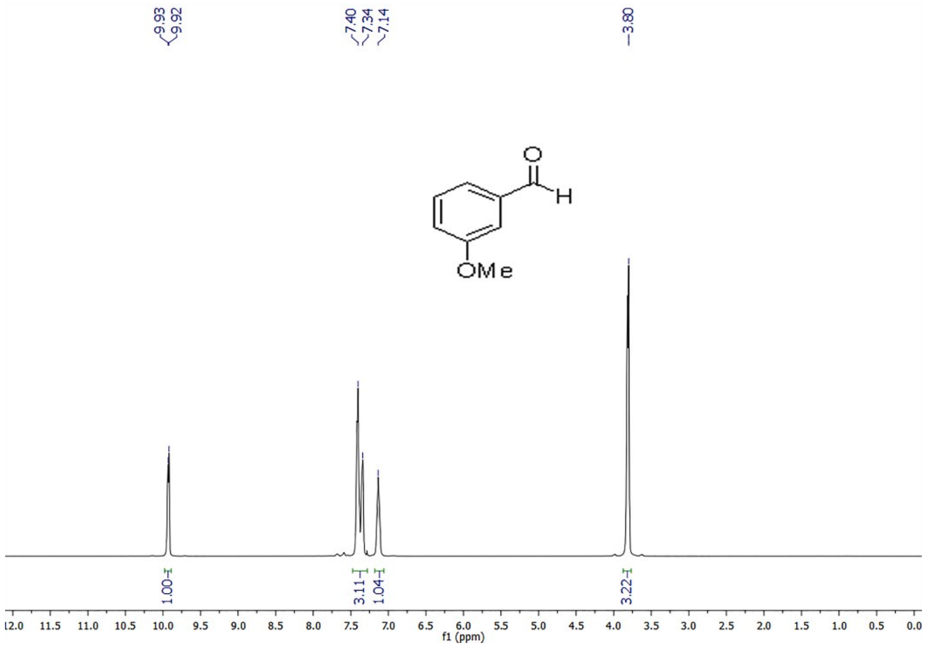
9.93
9.92
7.40
7.34
7.14
3.80
OMe
H
O
1.00
3.11
1.04
3.22
f1 (ppm)

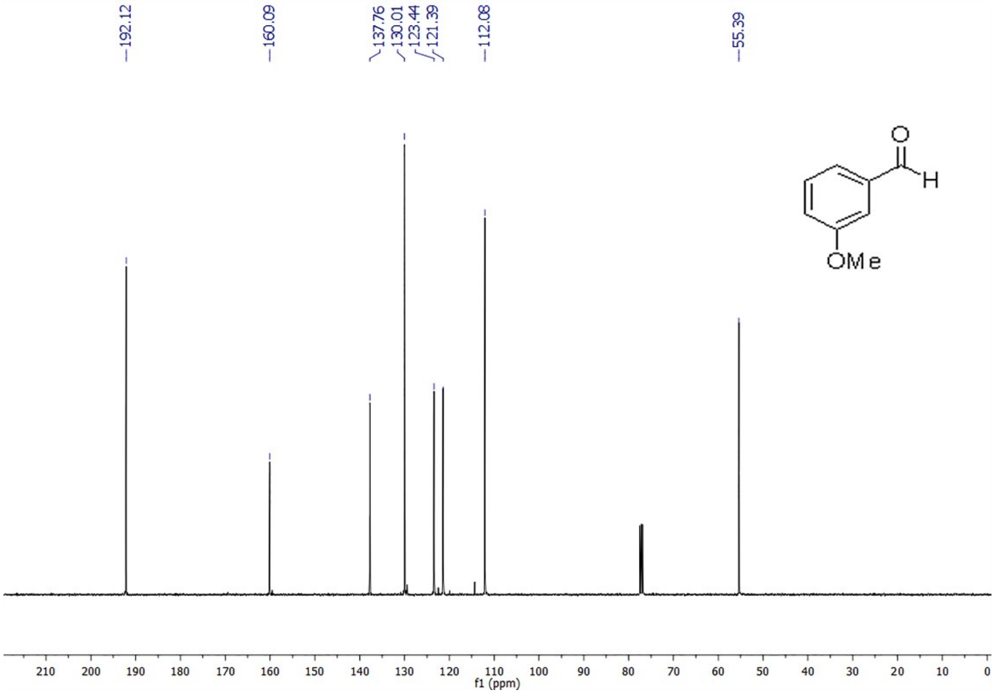
192.12
160.09
137.76
130.01
123.44
121.39
112.08
55.39
OMe
H
O
f1 (ppm)

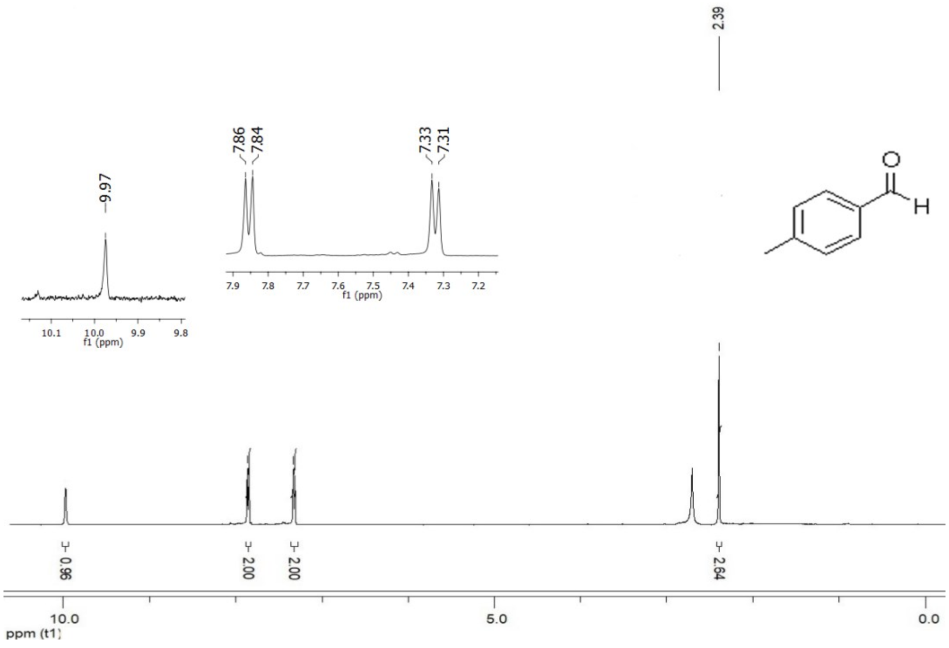
2.39
7.86
7.84
7.33
7.31
9.97
10.1
10.0
9.9
9.8
f1 (ppm)
7.9 7.8 7.7 7.6 7.5 7.4 7.3 7.2
H
O
0.96
2.00
2.00
2.64
10.0
5.0
0.0
ppm (t1)

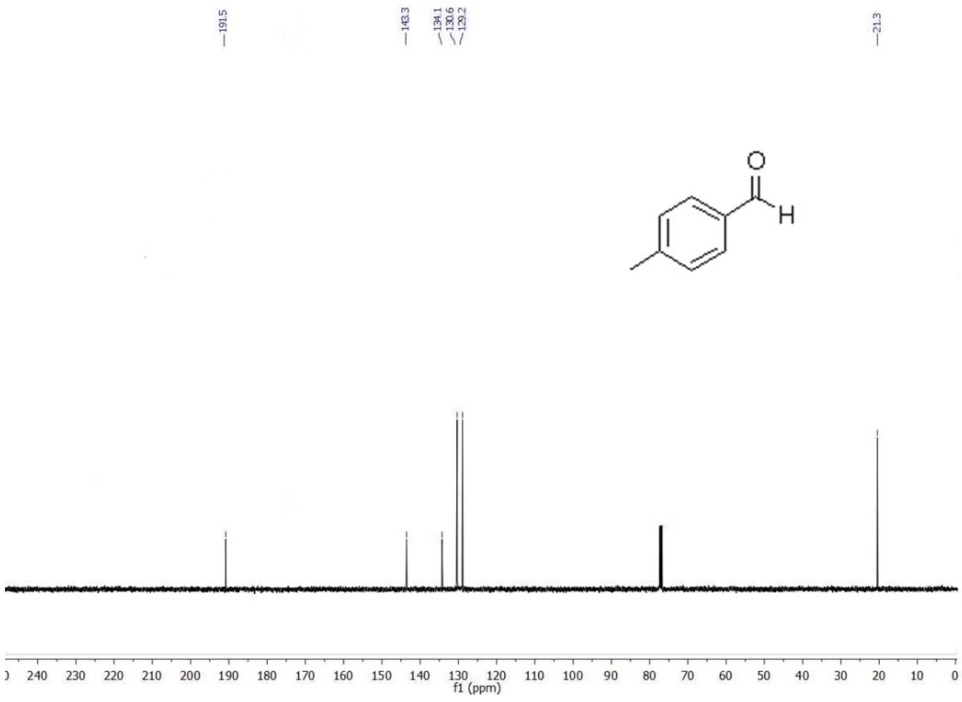
191.5
143.3
134.1
130.6
129.2
21.3
O
H
240 230 220 210 200 190 180 170 160 150 140 130 120 110 100 90 80 70 60 50 40 30 20 10 0
f1 (ppm)

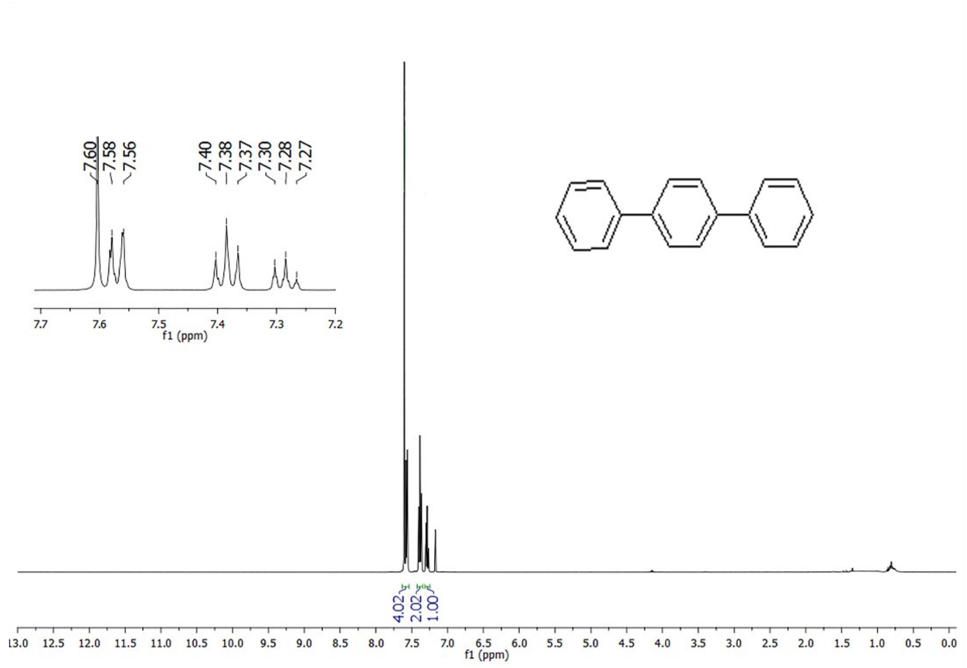
7.60
7.58
7.56
7.40
7.38
7.37
7.30
7.28
7.27
f1 (ppm)
4.02
2.02
1.00
f1 (ppm)

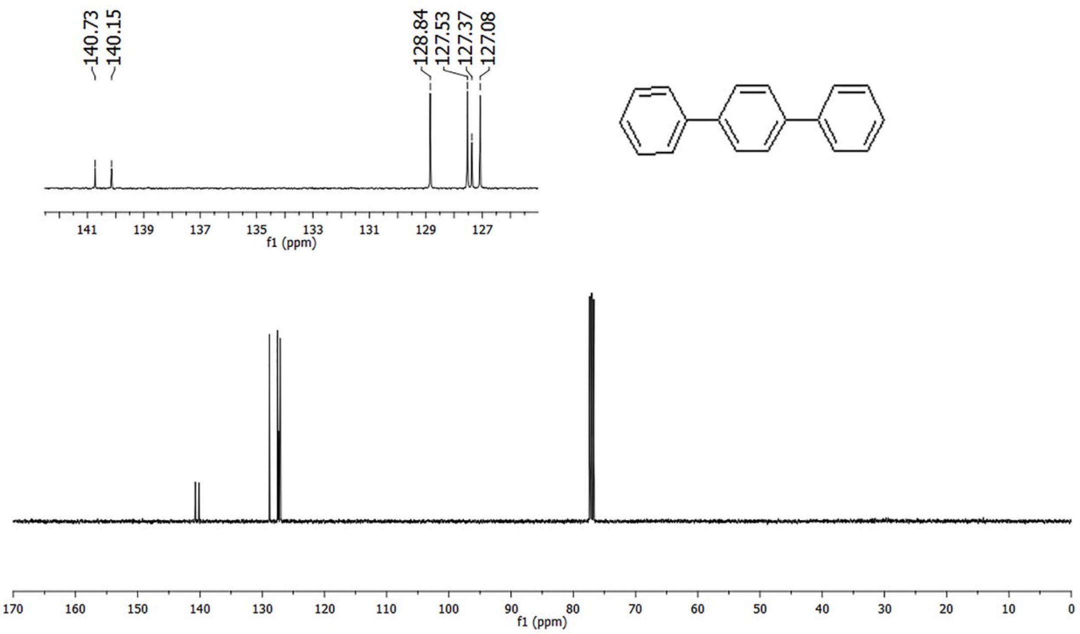
140.73
140.15
128.84
127.53
127.37
127.08
f1 (ppm)
f1 (ppm)

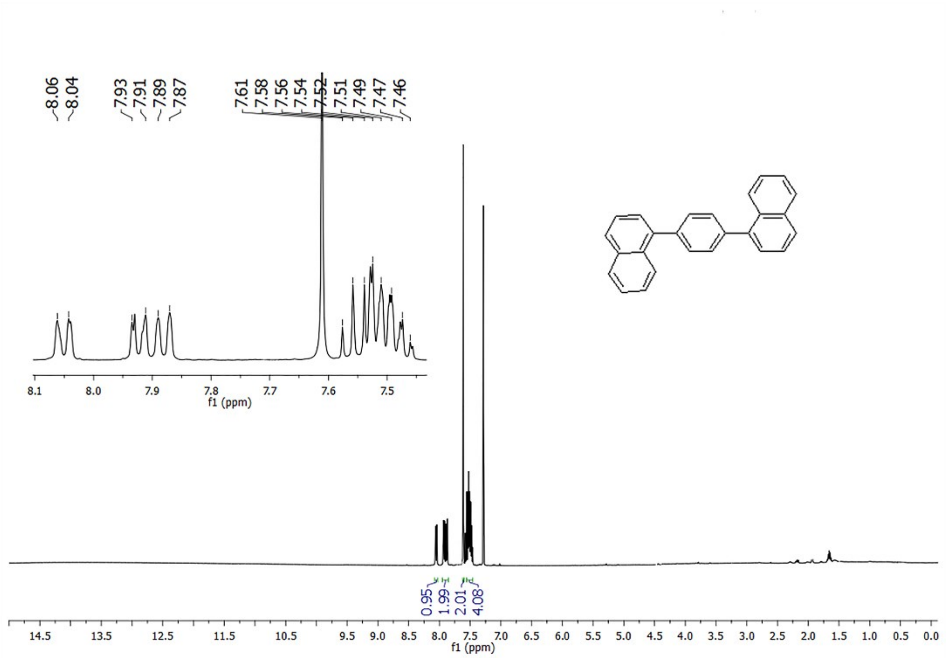
8.06
8.04
7.93
7.91
7.89
7.87
7.61
7.58
7.56
7.54
7.52
7.51
7.49
7.47
7.46
0.95
1.99
2.01
4.08
f1 (ppm)

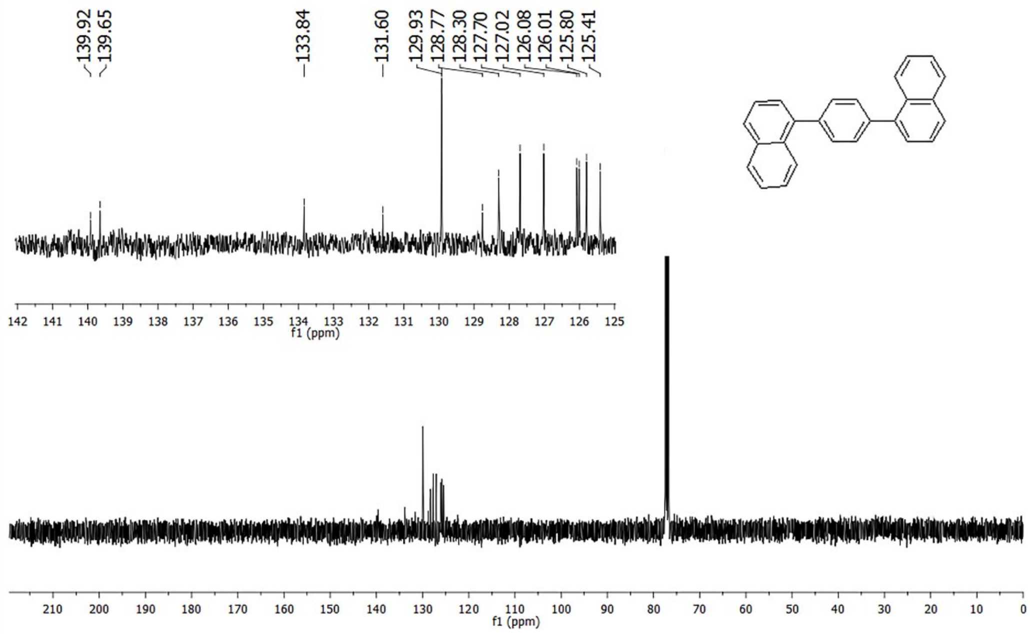
139.92
139.65
133.84
131.60
129.93
128.77
128.30
127.70
127.02
126.08
126.01
125.80
125.41
f1 (ppm)

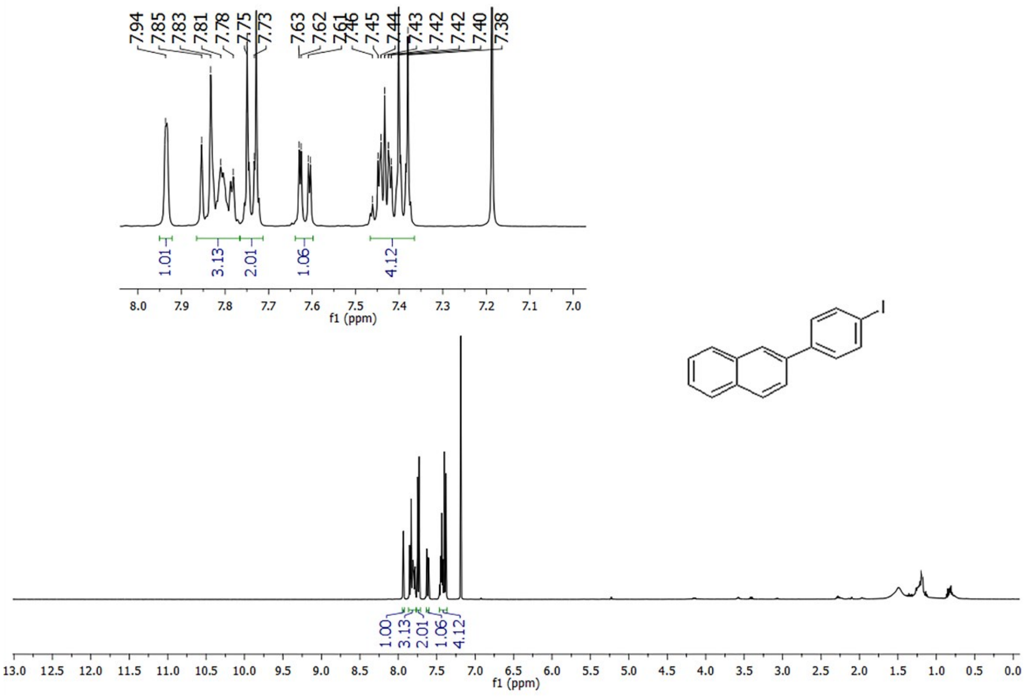
7.94
7.85
7.83
7.81
7.78
7.75
7.73
7.63
7.62
7.61
7.46
7.45
7.44
7.43
7.42
7.42
7.40
7.38
1.01
3.13
2.01
1.06
4.12
f1 (ppm)
I
1.00
3.13
2.01
1.06
4.12
f1 (ppm)

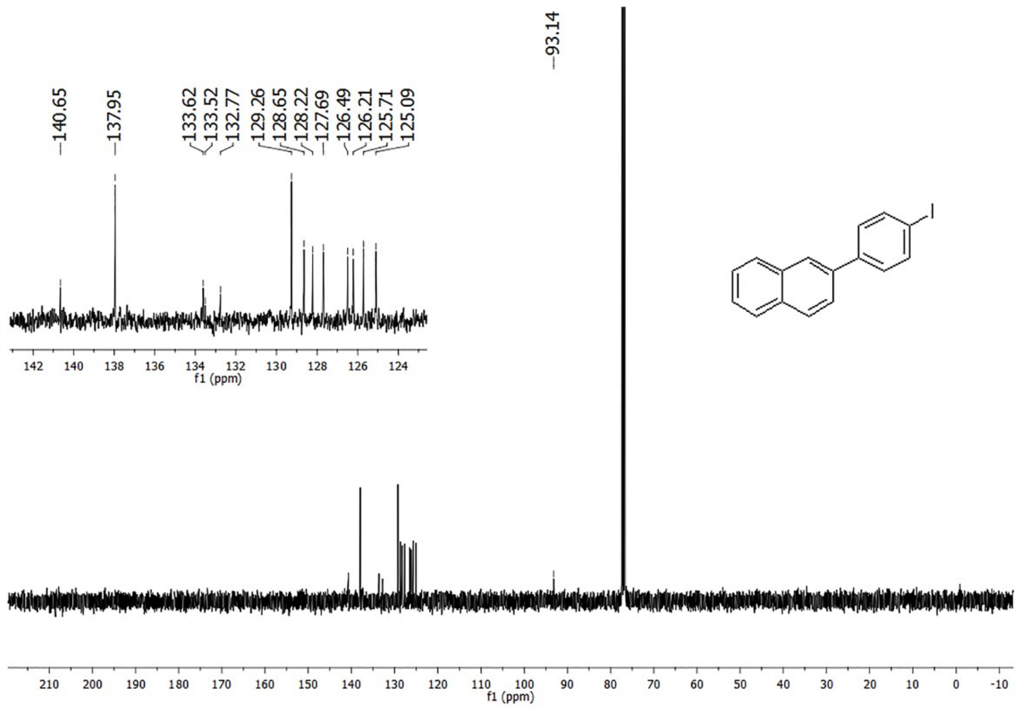
93.14
140.65
137.95
133.62
133.52
132.77
129.26
128.65
128.22
127.69
126.49
126.21
125.71
125.09
f1 (ppm)
I
f1 (ppm)

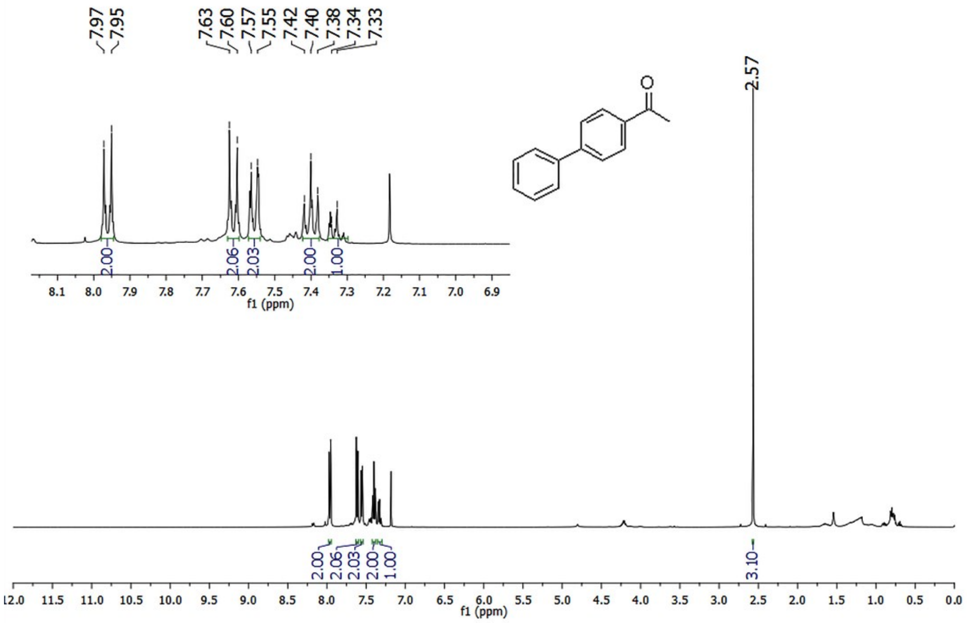
7.97
7.95
7.63
7.60
7.57
7.55
7.42
7.40
7.38
7.34
7.33
2.57
2.00
2.06
2.03
2.00
1.00
3.10
f1 (ppm)

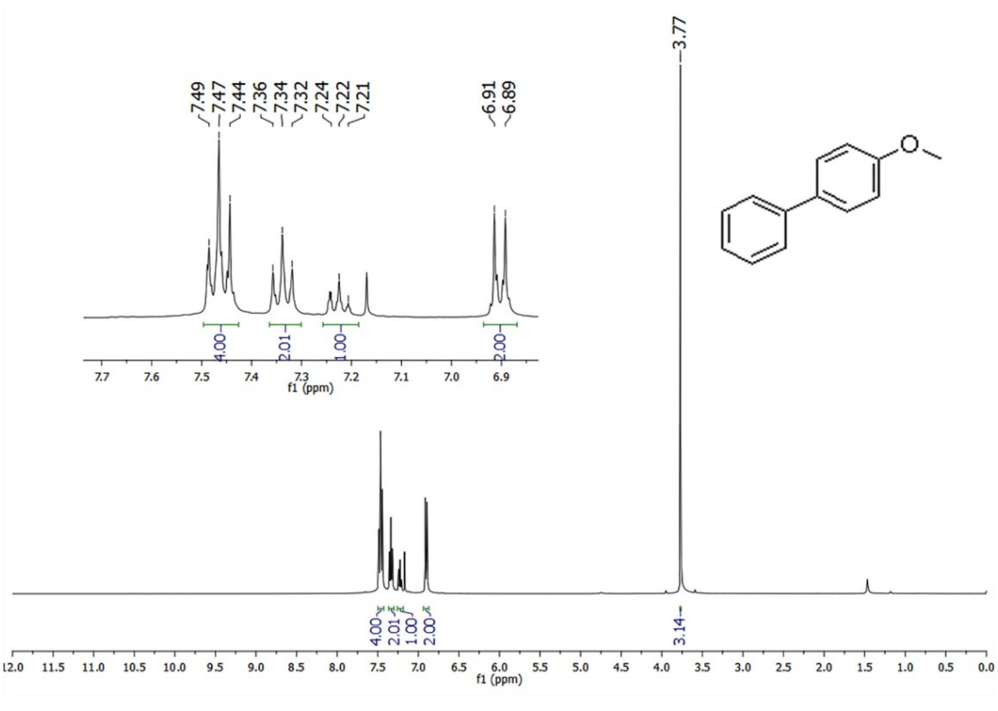
3.77
7.49
7.47
7.44
7.36
7.34
7.32
7.24
7.22
7.21
6.91
6.89
4.00
2.01
1.00
2.00
3.14
f1 (ppm)

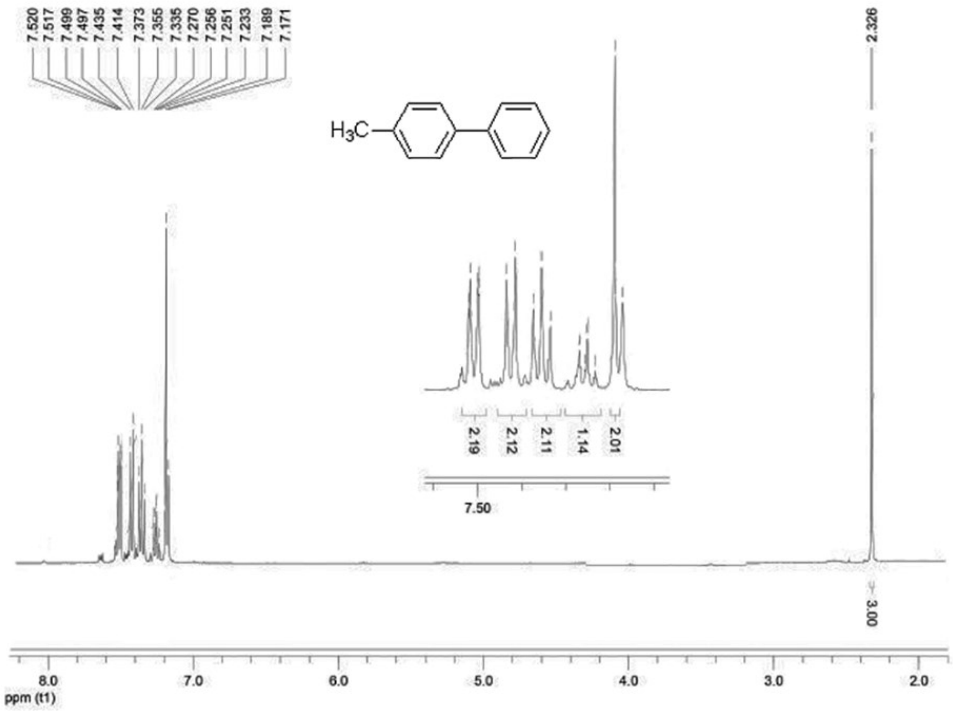
7.520
7.517
7.499
7.497
7.435
7.414
7.373
7.355
7.335
7.270
7.256
7.251
7.233
7.189
7.171
2.326
H_3C
2.19
2.12
2.11
1.14
2.01
7.50
3.00
8.0
7.0
6.0
5.0
4.0
3.0
2.0
ppm (t1)

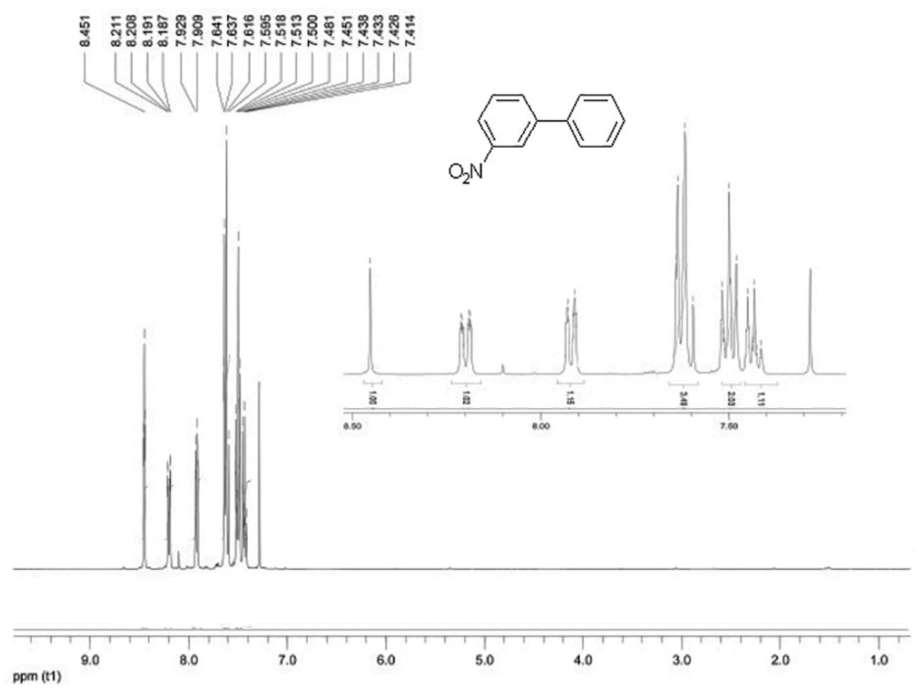
8.451
8.211
8.208
8.191
8.187
7.929
7.909
7.641
7.637
7.616
7.595
7.518
7.513
7.500
7.481
7.451
7.438
7.433
7.426
7.414
O_2N
8.50
8.00
7.50
9.0
8.0
7.0
6.0
5.0
4.0
3.0
2.0
1.0
ppm (t1)

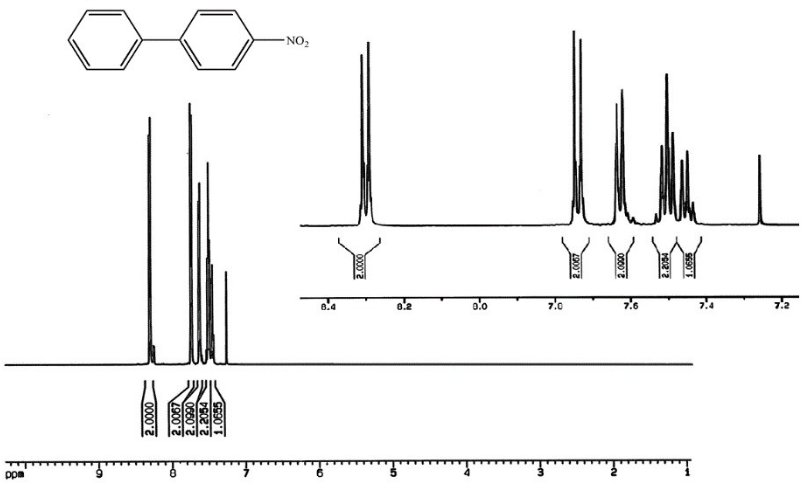
NO2
2.0000
2.0067
2.0960
2.2054
1.0655
ppm
9
8
7
6
5
4
3
2
1
8.4
8.2
8.0
7.8
7.6
7.4
7.2

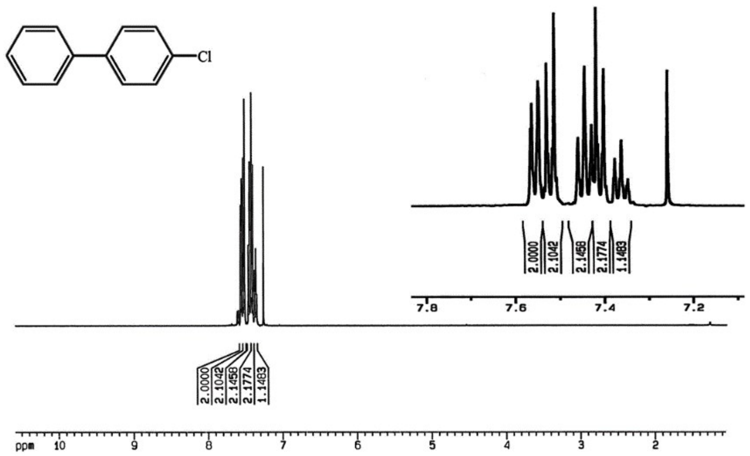
Cl
2.0000
2.1042
2.1458
2.1774
1.1483
7.8
7.6
7.4
7.2
ppm
10
9
8
7
6
5
4
3
2
